# Assessment of the Antioxidant and Photoprotective Properties of *Cornus mas* L. Extracts on HDF, HaCaT and A375 Cells Exposed to UVA Radiation

**DOI:** 10.3390/ijms252010993

**Published:** 2024-10-12

**Authors:** Martyna Zagórska-Dziok, Agnieszka Mokrzyńska, Aleksandra Ziemlewska, Zofia Nizioł-Łukaszewska, Ireneusz Sowa, Marcin Feldo, Magdalena Wójciak

**Affiliations:** 1 Department of Technology of Cosmetic and Pharmaceutical Products, Medical College, University of Information Technology and Management in Rzeszow, Sucharskiego 2, 35-225 Rzeszow, Poland; mzagorska@wsiz.edu.pl (M.Z.-D.); amokrzynska@wsiz.edu.pl (A.M.); aziemlewska@wsiz.edu.pl (A.Z.); zniziol@wsiz.edu.pl (Z.N.-Ł.); 2 Department of Analytical Chemistry, Medical University of Lublin, Aleje Raclawickie 1, 20-059 Lublin, Poland; i.sowa@umlub.pl; 3 Department of Vascular Surgery, Medical University of Lublin, Staszica 11 St., 20-081 Lublin, Poland; martinf@interia.pl

**Keywords:** *Cornus mas* L., UVA radiation, photoprotection, antioxidants, oxidative stress, tyrosinase inhibition, skin cells, melanoma

## Abstract

The influence of UV radiation on skin discoloration, skin aging and the development of skin cancer is widely known. As a part of this study, the effect of extracts from three varieties of *Cornus mas* L. (*C. mas* L.) on skin cells exposed to UVA radiation was assessed. The analyses were performed on both normal and cancer skin cells. For this purpose, the potential photoprotective effects of the obtained extracts (aqueous and ethanolic) was assessed by performing two cytotoxicity tests (Alamar blue and Neutral red). Additionally, the antioxidant capacity was compared using three different assays. The 2′,7′-dichlorodihydrofluorescein diacetate (H_2_DCFDA) probe was used to evaluate the intracellular level of free radicals in cells exposed to the simultaneous action of UVA radiation and dogwood extracts. Additionally, the ability to inhibit excessive pigmentation was determined by assessing the inhibition of melanin formation and tyrosinase activity. The obtained results confirmed the strong antioxidant properties of dogwood extracts and their photoprotective effect on normal skin cells. The ability to inhibit the viability of melanoma cells was also observed. Additionally, a reduction in oxidative stress in skin cells exposed to UVA radiation and a strong inhibition of melanin formation and tyrosinase activity have been demonstrated. This study shows that dogwood extract could be a valuable cosmetic raw material that can play both a photoprotective and antihyperpigmentation role in cosmetic preparations.

## 1. Introduction

UVA radiation (320–400 nm) constitutes 95% of the UV light reaching the Earth; therefore, it is radiation that can significantly affect the human body [1]. Exposure of the skin to solar radiation is associated with various skin changes, which include both skin disorders and impact on its aesthetic appearance [2]. Excessive exposure to UV radiation may result in atrophy, wrinkles, pigmentation changes, and the development of malignant skin tumors; the most common of which are basal cell carcinoma, squamous cell carcinoma and malignant melanoma [3]. The negative impact of this radiation on the appearance and condition of the skin is related to its impact on the metabolism of skin cells and the increase of the amount of free radicals causing oxidative stress. This may result in damage to the main building proteins of the skin, such as collagen and elastin [2,4]. This radiation also affects the activity of skin enzymes [2]. UV radiation can increase the activity of matrix metalloproteinases (MMPs) such as MMP-1, MMP-3 and MMP-9, which results in skin photoaging due to the degradation of proteins such as collagen, elastin, fibronectin and proteoglycans [2,5]. Repeated exposure to UVA radiation also affects changes in the activity of cathepsins (B, D, K and G) in the skin and can intensify its photoaging, weaken the immune response and disrupt the processes of proliferation and keratinization. Changes in the activity of cathepsin B can additionally affect matrix degradation and cell invasion [2,6,7]. UV radiation can also increase the activity of tyrosinase, which may result in increased melanin production [8]. UVA radiation, compared to UVB radiation, is absorbed to a lesser extent by nucleotides, which results in less direct DNA damage [9]. However, this radiation can be absorbed by other molecules present in the skin that are cellular chromophores such as urocanic acid, proteins containing aromatic amino acids, as well as melanins and their precursors [10]. This leads to oxidative damage to cellular structures resulting from the production of photoexcited species such as singlet oxygen and free radicals [11]. Due to the fact that UVA radiation can affect both the dermis and the epidermis, it is mainly responsible for photoaging of the skin [12]. This radiation may affect the expression of fibroblast genes, resulting in changes in their secretory capacity, senescence and apoptosis [12,13]. This radiation also affects the ultrastructure and function of the epidermis by changing the thickness of the skin and disturbing its barrier capacity. Additionally, it may result in increased water loss from the epidermis, poorer hydration and poorer ability to renew the epithelium during the wound healing process [14,15].

Due to various side effects of synthetic photoprotective ingredients used in cosmetic preparations, manufacturers are looking for alternative chemical compounds of natural origin [16]. These phytochemicals, in addition to their multidirectional biological activity, can effectively protect against the harmful effects of UV radiation [17]. Natural photoprotective compounds can reduce the damage caused by this radiation and modulate various cellular pathways in cells. These compounds are also able to reduce inflammation and eliminate intracellular oxidative stress [18]. This may result in inhibition or changes in the promotion and progression of cancer resulting from excessive exposure to UV radiation [19]. Natural photoprotectors are used primarily in preparations applied directly to the skin and are mainly responsible for absorbing, reflecting or scattering rays reaching the skin surface [20]. Chemical filters approved for use in cosmetic preparations can interact with the solvents and matrices used in sunscreen formulations, which in turn can lead to cross-sensitization [21]. As is well known, plant-based photoprotective compounds are often characterized by a broad spectrum of radiation absorption (in the range of 200 to 400 nm) and a strong antioxidant potential, which is the reason why they are increasingly used in cosmetic formulations as supportive agents in UV-protective skin preparations [22]. *Cornus mas* L. (*C. mas* L.) is a plant with a wide biological activity, which allows it to be used in cosmetic preparations with multidirectional effects [23,24,25]. Dogwood fruit extracts are characterized by high antioxidant capacity, antimicrobial potential and cytoprotective properties [25]. These extracts can also inhibit the activity of various enzymes including α-glucosidase, acetylcholinesterase, cyclooxygenase-2, elastase and collagenase [23,26]. *C. mas* L. fruits also have anti-inflammatory properties due to their ability to influence the level of anti- and pro-inflammatory interleukins such as IL-1β, IL-6, IL-8, IL-10, IL-13 and TNF-α [26,27,28]. Additionally, extracts from *C. mas* L. have a positive effect on the skin by increasing its hydration and inhibiting transepidermal water loss [24]. However, so far there have been no literature reports regarding the photoprotective properties of this plant. The wide spectrum of phytochemicals in dogwood fruits indicates their great potential in protection against UV radiation, so the aim of this study was to assess the photoprotective properties of these fruits. Many studies indicate that compounds identified in *C. mas* L. such as galloyl hexoside, gallic acid, loganic acid, quinic acid or quercetin 3-glucuronide exhibit photoprotective activity [29,30,31,32]. For this purpose, the effect of different types of fruit extracts from three dogwood varieties on normal and cancerous skin cells exposed to UVA radiation was assessed. Additionally, their antioxidant properties were compared and the impact on the intracellular level of free radicals in keratinocytes, fibroblasts and melanoma cells after irradiation was determined. Their effect on melanin formation and tyrosinase activity was also evaluated in order to determine their ability to protect against the formation of hyperpigmentation disorders on the skin.

## 2. Results and Discussion

### 2.1. Quantitative Determination of Components Found in the Extracts from Fruit of C. mas *L.*

Ultra-performance liquid chromatography (UPLC) was applied to separate the components of different extracts from the yellow, red, and dark red fruit of *C. mas* L. varieties (Figure S1). The compounds were tentatively identified based on spectral and MS data (Table S1) and comparison with literature reports [33,34]. Identification was also confirmed by standards when they were commercially available.

Table 1 summarizes the results of quantification expressed as µg/mL of the extracts.

The chromatographic analysis showed statistically significant differences in the concentration of the components between different types of extracts. In general, the concentration of phenolics was higher in alcoholic extracts than in water extracts. This trend highlights the efficacy of adding ethanol to the extraction solvent for maximizing the yield of phenolics, as confirmed by previous research [26]. The positive effect of organic solvents on the effectiveness of isolating flavonoids and polyphenolic acids was also observed in other plant materials [35,36]. It has been found that alcohol–water mixtures have higher mass transfer velocities, reduced viscosity and surface tension, and therefore enhance penetration into the plant material. Moreover, the solubility of polyphenolic compounds in such solvents is usually increased [37].

An exception to this pattern was observed with gallic acid derivatives, which were found in higher abundance in water extracts, consistent with data reported in the literature [24]. The reports confirm that highly polar glycoside derivatives are less soluble in organic solvents [38]. Interestingly, quinic acid showed a similar concentration across all extracts, regardless of the solvent used, suggesting a uniform solubility profile for this compound. Differences in quantitative composition were also observed between varieties. As previously reported [24], loganic acid, belonging to the iridoid class, was the predominant constituent of the tested extract. Its highest concentration was found in the 30% ethanol extract from the yellow-fruit variety (278.0 µg/mL), followed by the dark ruby-red variety (151.0 µg/mL). Klymenko et al. also reported that yellow *C. mas* L. fruits are the most abundant in iridoid compounds [39].

Quinic acid, gallic acid derivatives (galloyl hexoside and galloyl-*d*-sedoheptulose) and quercetin 3-glucuronide were also the most abundant in yellow and dark ruby-red fruit varieties. Similar to Klymenko et al., kaempferol 3-*O*-galactoside was not detected in the yellow fruit variety. Moreover, in yellow fruit extracts, no anthocyanins were detected, and the highest content was noted in the extracts from dark ruby-red fruits. This suggests that the color of the fruit is directly linked to the anthocyanin concentration, with darker-colored fruits containing more of these pigment compounds.

### 2.2. Determination of Antioxidant Properties

#### 2.2.1. Assessment of Antioxidant Capacity Using ABTS and DPPH Radicals and the FRAP Assay

The antioxidant properties of phytochemicals contained in plant materials are the result of different mechanisms of action. Therefore, tests such as ABTS, DPPH and FRAP were used in assessing the antioxidant properties of the fruits of three dogwood varieties. These assays allowed the evaluation of antioxidant capacity based on both hydrogen atom transfer and single electron transfer. Very often, these two types of reactions occur simultaneously, and the dominant mechanism is mainly determined by the structure, solubility, polarity and partition coefficient of the antioxidant compound [39,40]. The studies conducted as a part of this study assessed the antioxidant potential of nine different dogwood fruit extracts. For this purpose, the ability to scavenge synthetic free radicals (DPPH and ABTS) and reduce Fe^3+^ ions to Fe^2+^ ions was assessed using the FRAP method. Additionally, the influence of the tested extracts on the intracellular level of free radicals in normal and cancer skin cells exposed to UVA radiation was also assessed. Antioxidant protection is extremely important in this case, as UV radiation may have an immunosuppressive effect, which accelerates the skin aging process and may also result in increased carcinogenesis [41]. Research shows that plant extracts are a significant source of antioxidants, which are helpful in preventing the progression of various oxidative damage. Antioxidants can neutralize various types of free radicals, such as hydroxyl radicals, superoxide anion radicals and even lipid radicals [42]. By reducing peroxide and alkoxy radicals, they protect lipids by preventing the formation of lipid peroxidation products. Plant compounds with antioxidant properties protect cell cement lipids against oxidation, can influence the activity of various enzymes, stimulate skin microcirculation and improve the condition of blood vessels [41,42]. Additionally, they delay skin aging processes, absorb UVA and UVB radiation, and eliminate skin discoloration [43].

**Figure 1:**
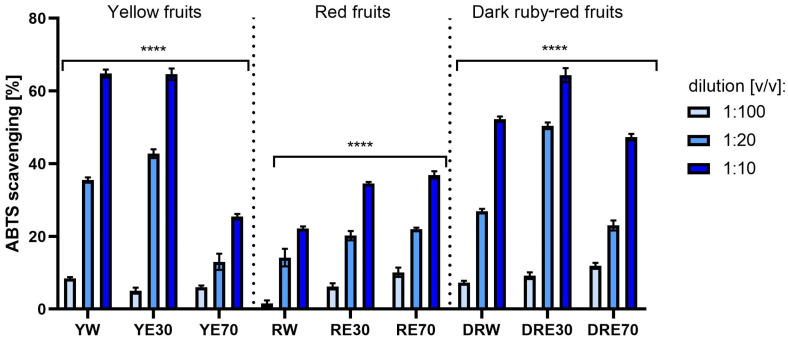
The effect of extracts from three varieties of *Cornus mas* L. (with yellow, red and dark ruby-red fruits) on ABTS radical scavenging. Analyses were performed for water and two water–ethanolic extracts (30 and 70% (*v*/*v*)) at a dilution of 1:100, 1:20, and 1:10 (*v*/*v*). The research used yellow fruit extracts (water (YW), water–ethanol 30:70 (YE30), water–ethanol 70:30 (YE70)), red fruit extracts (water (RW), water–ethanol 30:70 (RE30), water–ethanol 70:30 (RE70)) and dark ruby-red extracts (water (DRW), water–ethanol 30:70 (DRE30), water–ethanol 70:30 (DRE70)). Data represent mean ± SD of three independent experiments in which each sample was tested in triplicate. **** *p* < 0.0001.

**Figure 2:**
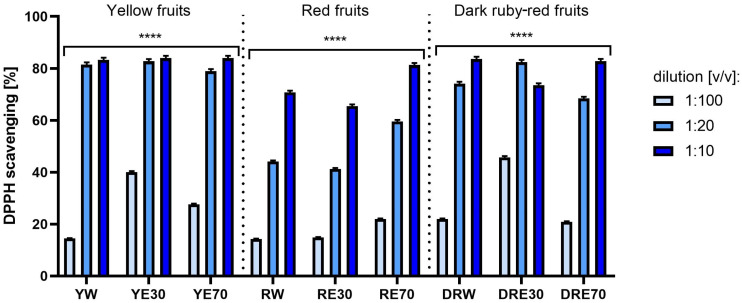
The effect of extracts from three varieties of *Cornus mas* L. (with yellow, red and dark ruby-red fruits) on DPPH radical scavenging. Analyses were performed for water and two water–ethanolic extracts (30 and 70% (*v*/*v*)) at a dilution of 1:100, 1:20, and 1:10 (*v*/*v*). The research used yellow fruit extracts (water (YW), water–ethanol 30:70 (YE30), water–ethanol 70:30 (YE70)), red fruit extracts (water (RW), water–ethanol 30:70 (RE30), water–ethanol 70:30 (RE70)) and dark ruby-red extracts (water (DRW), water–ethanol 30:70 (DRE30), water–ethanol 70:30 (DRE70)). Data represent mean ± SD of three independent experiments in which each sample was tested in triplicate. **** *p* < 0.0001.

Analyses assessing the ability to neutralize DPPH and ABTS free radicals indicated an antioxidant effect dependent on the dose and type of extract. In the case of all nine extracts, the free radical scavenging potential increased statistically significantly with decreasing dilution. In both tests, a stronger effect of extracts from yellow and dark red dogwood fruit was observed compared to extracts from red fruit. The antioxidant properties of the tested extracts also differed depending on the extractant used during the extraction process. The strongest potential for scavenging the ABTS radical was found in water (YW) and water–ethanol extracts (YE30, 70:30 (*v*/*v*)) from yellow fruits and water-ethanol extracts (DRE30, 70:30 (*v*/*v*)) from dark ruby-red fruits, which neutralized over 60% of these radicals. In the case of the DPPH assay, no such large differences in antioxidant capacity were observed between extracts obtained using different extraction media. All types of extracts from yellow and dark red fruits in the lowest tested dilution scavenged over 80% of DPPH radicals (Figure 1 and Figure 2).

**Figure 3:**
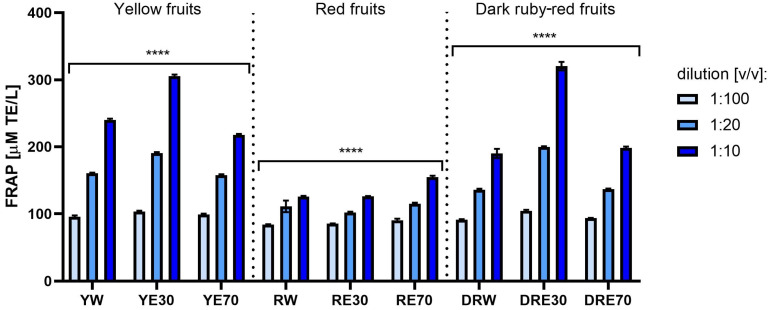
Antioxidant capacity in FRAP assay of extracts from three varieties of *Cornus mas* L. (with yellow, red and dark ruby-red fruits). Analyses were performed for water and two water–ethanolic extracts (30 and 70% (*v*/*v*)) at a dilution of 1:100, 1:20, and 1:10 (*v*/*v*). The research used yellow fruit extracts (water (YW), water–ethanol 30:70 (YE30), water–ethanol 70:30 (YE70)), red fruit extracts (water (RW), water–ethanol 30:70 (RE30), water–ethanol 70:30 (RE70)) and dark ruby-red extracts (water (DRW), water–ethanol 30:70 (DRE30), water–ethanol 70:30 (DRE70)). The results were expressed in Trolox equivalents (µmol TE/L). Data represent mean ± SD of three independent experiments in which each sample was tested in triplicate. **** *p* < 0.0001.

The FRAP method also showed a concentration-, variety- and extractant-dependent reduction potential of the tested *C. mas* L. extracts. The results obtained in this assay were expressed as Trolox micromolar equivalent per liter (µM TE/L). In this test, the strongest antioxidant capacity of extracts obtained from varieties with yellow and dark ruby-red fruits was also observed. The strongest reduction of iron ions was obtained for water-ethanol extracts with 30% ethanol content; for which, in the case of the yellow fruit extract, the antioxidant capacity was 305.49 ± 2.16 µM TE/L and for the dark ruby-red fruit extract it was 320.33 ± 6.31 µM TE/L (Figure 3). Taking into account the results of the three tests described above, it can be concluded that the strongest antioxidant potential is demonstrated by the 30% water–ethanol extract from yellow fruits (YE30) and dark red fruits (DRE30), for which the strongest reducing activity was recorded.

The antioxidant capacity of the tested extracts is certainly the result of the action of biologically active compounds present in dogwood fruits, some of which were confirmed in chromatographic analyses. Gallic acid and its derivatives may play an important role in this case. Lu et al., in their analyses on a model system involving the incorporation of a stable DPPH free radical into the lipid membrane, indicated the antioxidant capacity of these compounds. These authors noted that the ability to reduce these radicals by gallic acid derivatives is strictly dependent on the presence of phenolic hydroxyl groups, and the methylation of these groups and the increase in the length of the acyl chain are associated with a decrease in the antioxidant potential. Interestingly, these authors did not observe a positive correlation between the hydrophobicity of the tested compounds and the ability to scavenge the lipophilic DPPH radical in liposomes. However, they noted that the hydrophobicity of compounds plays an extremely important role in antiradical activity in cellular systems, where too low hydrophobicity of compounds may significantly inhibit their penetration through cell membranes [44].

The antioxidant effect may also be the result of the action of iridoids present in dogwood fruits, mainly loganic acid. In vitro radical scavenging analyses conducted by Abirami et al. showed strong antioxidant capacity of this compound in terms of scavenging the DPPH radical, superoxide radicals and hydroxyl radicals. Moreover, these authors also indicated that loganic acid inhibited lipid peroxidation and intracellular ROS production caused by the toxic effects of heavy metals [45]. Dzydzan et al. also confirmed the antioxidant effect of this acid, indicating its ability to increase the activity of catalase, glutathione peroxidase and glutathione reductase, and reduce the intracellular ROS level [46]. Shi et al. confirmed the antioxidant capacity of another iridoid (cornuside), which we also identified in our extracts. These authors showed that this compound can affect the activity of superoxide dismutase and glutathione peroxidase [47].

Phenolic acids also play an extremely important role in the antioxidant capacity of plant materials. Compounds such as quinic acid, protocatechuic acid, caftaric acid and chlorogenic acid identified in dogwood fruits are compounds with antioxidant properties, as proven in numerous works [48,49,50,51,52]. The mechanism of the antioxidant capacity of this group of compounds is closely related to their structure, in which the benzene ring plays an important role, as well as the number and position of various functional groups, primarily hydroxyl groups [53]. The action of these compounds may be based on the transfer of a hydrogen atom, a single electron or sequential electron transfer, with the loss of protons [53,54,55]. An important mechanism of phenolic acid’s action is also the chelation of transition metals such as iron, which results in the formation of stable products and a reduction in the formation of reactive free hydroxyl radicals during the Fenton reaction [56]. Chelation reactions can be catalyzed by various transition metals, such as copper, cobalt or manganese [53,57,58]. This chelation is strictly dependent on the reduction potential and structure of phenolic compounds [53]. Phenolic acids can also directly or indirectly affect the activation or expression of various antioxidant signaling pathways. They can affect the Keap1-Nrf2 pathway, which is a cellular defense system against excessive oxidative stress in cells [59]. The effect of this is the reduction of the level of malondialdehyde by regulating the expression of glutathione and various endogenous antioxidant enzymes. These enzymes are mainly superoxide dismutase, glutathione peroxidase and catalase [51,60,61].

The antioxidant mechanism of action of anthocyanins identified in red and dark ruby-red fruits of *C. mas* L., such as cyanidin 3-*O*-galactoside or pelargonidin 3-*O*-glucoside, is also probably based on the previously mentioned mechanisms of hydrogen atom donation or single electron transfer [62]. This activity may also be related to the ability of this group of compounds to neutralize the initiators of the oxidation process, by absorbing photons, chelating metal ions such as Fe^3+^ or Cu^2+^, inhibiting lipid oxidation and neutralizing ROS [62,63,64]. Anthocyanins may also exert indirect antioxidant effects by inducing intracellular Nrf2 activation and expression of various antioxidant genes [65]. The antioxidant capacity of this group of compounds is closely dependent on their chemical structure. The number and position of hydroxyl groups, conjugation groups, as well as the degree of glycosylation and the presence of donating electrons in the flavonoid ring structure play an important role [66,67]. The ability to inhibit excessive free radical production by anthocyanins may also be related to the inhibition of the activity of different enzymes and sequestration of trace elements that are involved in the production of free radicals [68]. In addition, flavonoids can reduce oxidative stress by donating hydrogen atoms to the superoxide radical, forming a flavonoid radical, and inhibiting membrane lipid peroxidation [66,69]. Flavonoids also have a multidirectional effect in the context of antioxidant defense, as they have the ability to scavenge free radicals and chelate transition metal ions [70,71]. This group of compounds can also inhibit the activity of enzymes associated with the production of excessive amounts of free radicals such as cyclooxygenase, xanthine oxidase, protein kinase C, lipoxygenase, microsomal monooxygenase, and mitochondrial succinyl oxidase as well as NADPH oxidase [70,72]. Flavonoids also have the ability to stimulate intracellular antioxidant enzymes such as phase II metabolizing enzymes, which include UDP-glucuronosyltransferases, glutathione *S*-transferases, sulfotransferases, N-acetyltransferases as well as methyltransferases [70,72]. As in the case of the previously described groups of compounds, also in the case of flavonoids, there is a high correlation between their antioxidant capacity and structure. An important role is played by the number and position of the hydroxyl group, the ortho-dihydroxyl system in the B ring, the unsaturated C2-C3 bond combined with C-4 carbonyl group in the C skeleton and *O*-methylation [73,74].

#### 2.2.2. Determination of Intracellular Levels of Reactive Oxygen Species (ROS)

In order to more comprehensively assess the antioxidant properties of the tested samples, in addition to the previously described chemical methods, an assessment of the reduction of oxidative stress in cellular systems was carried out. Excessive accumulation of reactive oxygen species in cells causes oxidative stress, which negatively affects the functioning of cells and the structure of cell membranes, lipids, proteins, lipoproteins and deoxyribonucleic acid [75]. Therefore, it is extremely important to restore the balance between the production and neutralization of free radicals after the exposure of cells to pro-oxidant factors [76].

**Figure 4:**
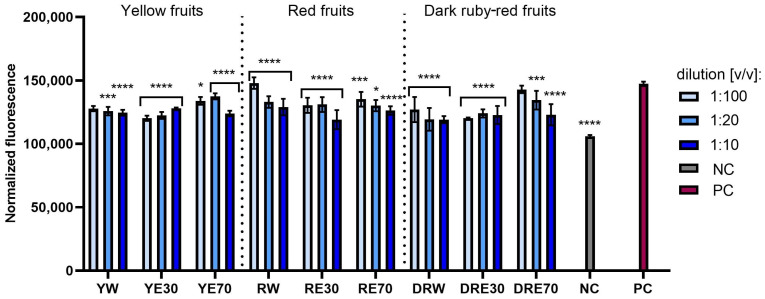
The effect of extracts from three varieties of *Cornus mas* L. (with yellow, red and dark red fruits) on the intracellular level of reactive oxygen species in fibroblasts (HDF) exposed to UVA radiation (5 J/cm^2^ for 1 h). The positive control (PC) are HDF cells treated with UVA radiation (without the addition of extracts), and the negative control (NC) are cells exposed to neither UVA radiation nor extracts. Analyses were performed for water and two water–ethanolic extracts (30 and 70% (*v*/*v*)) at a dilution of 1:100, 1:20, and 1:10 (*v*/*v*). The research used yellow fruit extracts (water (YW), water–ethanol 30:70 (YE30), water–ethanol 70:30 (YE70)), red fruit extracts (water (RW), water–ethanol 30:70 (RE30), water–ethanol 70:30 (RE70)) and dark ruby-red extracts (water (DRW), water–ethanol 30:70 (DRE30), water–ethanol 70:30 (DRE70)). The exposure time to the extracts was 24 h. Data represent mean ± SD of three independent experiments in which each sample was tested in triplicate. **** *p* < 0.0001, *** *p* < 0.001, * *p* < 0.05.

**Figure 5:**
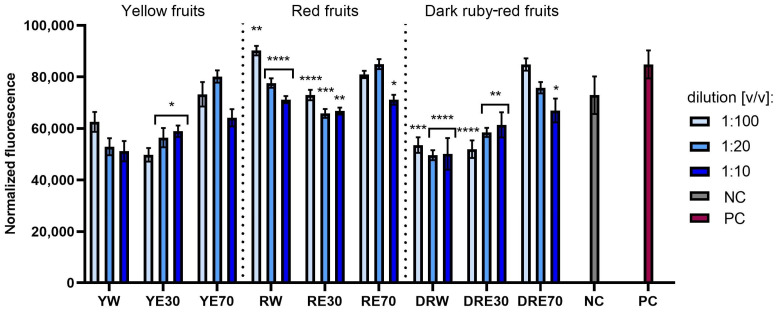
The effect of extracts from three varieties of *Cornus mas* L. (with yellow, red and dark red fruits) on the intracellular level of reactive oxygen species in keratinocytes (HaCaT) exposed to UVA radiation (5 J/cm^2^ for 1 h). The positive control (PC) are HaCaT cells treated with UVA radiation (without the addition of extracts), and the negative control (NC) are cells exposed to neither UVA radiation nor extracts. Analyses were performed for water and two water–ethanolic extracts (30 and 70% (*v*/*v*)) at a dilution of 1:100, 1:20, and 1:10 (*v*/*v*). The research used yellow fruit extracts (water (YW), water–ethanol 30:70 (YE30), water–ethanol 70:30 (YE70)), red fruit extracts (water (RW), water–ethanol 30:70 (RE30), water–ethanol 70:30 (RE70)) and dark ruby-red extracts (water (DRW), water–ethanol 30:70 (DRE30), water–ethanol 70:30 (DRE70)). The exposure time to the extracts was 24 h. Data represent mean ± SD of three independent experiments in which each sample was tested in triplicate. **** *p* < 0.0001, *** *p* < 0.001, ** *p* < 0.01, * *p* < 0.05.

**Figure 6:**
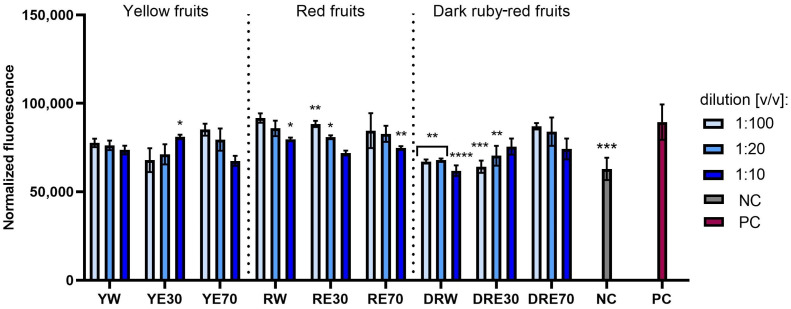
The effect of extracts from three varieties of *Cornus mas* L. (with yellow, red and dark red fruits) on the intracellular level of reactive oxygen species in melanoma cells (A375) exposed to UVA radiation (5 J/cm^2^ for 1 h). The positive control (PC) are A375 cells treated with UVA radiation (without the addition of extracts), and the negative control (NC) are cells exposed to neither UVA radiation nor extracts. Analyses were performed for water and two water–ethanolic extracts (30 and 70% (*v*/*v*)) at a dilution of 1:100, 1:20, and 1:10 (*v*/*v*). The research used yellow fruit extracts (water (YW), water–ethanol 30:70 (YE30), water–ethanol 70:30 (YE70)), red fruit extracts (water (RW), water–ethanol 30:70 (RE30), water–ethanol 70:30 (RE70)) and dark ruby-red extracts (water (DRW), water–ethanol 30:70 (DRE30), water–ethanol 70:30 (DRE70)). The exposure time to the extracts was 24 h. Data represent mean ± SD of three independent experiments in which each sample was tested in triplicate. **** *p* < 0.0001, *** *p* < 0.001, ** *p* < 0.01, * *p* < 0.05.

This study compared the tested extracts in terms of their ability to reduce oxidative stress in cells exposed to UVA radiation. Analyses conducted on normal (fibroblasts and keratinocytes) and cancerous (melanoma) skin cells indicated that the exposure of these cells to UVA radiation causes an increase in the intracellular level of free radicals (ROS) (Figure 4, Figure 5 and Figure 6). However, pre-treatment of these cells with the tested dogwood extracts results in a decrease in the ROS level in all types of cells tested. This effect depends on the dogwood variety, concentration and solvents used during extraction. In the case of both normal and cancer cells, the strongest antioxidant capacity was recorded for extracts from yellow and dark ruby-red fruits. Comparing the extraction media used, it was found that the level of free radicals in cells is reduced the least by water–ethanol extracts with 70% ethanol content (in the case of all three varieties).

In our previous work, we demonstrated the ability of dogwood extracts to reduce oxidative stress in normal skin cells exposed to the pro-oxidant hydrogen peroxide [26]. Reducing the ROS level in fibroblasts and keratinocytes protects these cells from loss of normal activity and structural changes, allowing them to maintain their functions [77].

ROS play an important role in cancer cells because they affect the tumor microenvironment and the ability to metastasize. Additionally, these molecules are also involved in the process of transformation and disease progression. It should also be noted that chronic oxidative stress also significantly increases the susceptibility of melanocytes to oncogenic transformation. The particular sensitivity of melanoma to oxidative stress is also related to the involvement of melanin synthesis in the formation of ROS in cells [78,79].

The ability to reduce the intracellular ROS level in the tested cells is probably related to the content of many antioxidant compounds in dogwood fruits, the activity of which may depend on both the structure and the mechanism of action. Lu et al., in their research, indicated that the activity of gallic acid and its derivatives in eliminating various cellular damages caused by oxidative stress is the result of their ability to scavenge free radicals and is strictly dependent on the hydrophobicity of the molecules of these compounds. This hydrophobicity allows for more efficient penetration of cell membranes, which allows reaching of those places in cells where the accumulation of free radicals is the highest [44]. To sum up, the antioxidant properties and the reduction of intracellular oxidative stress in the tested cells are certainly based on many of the above-described mechanisms of antioxidant action of various groups of biologically active compounds present in dogwood fruit extracts.

The applied assay using the 2′,7′-dichlorodihydrofluorescein diacetate (H_2_DCFDA) probe has a number of advantages, such as the ability to penetrate cells, high sensitivity to changes in the redox state of the cell, simplicity and the ability to kinetically monitor intracellular ROS changes [8]. Although the compound was initially considered a specific H_2_O_2_ indicator, it is now known that it is oxidized by other ROS, such as HO^•^ and ROO^•^ and by RNS (reactive nitrogen species), including ^•^NO and ONOO^−^ [80]. On the other hand, this method has various limitations that should be taken into account when designing the experiment. It is important to select the optimal probe concentration by assessing the initial background, which helps to avoid overestimation of the fluorescence signal [81]. When conducting the analyses using cell lines, it should also be taken into account that this compound is sensitive to the presence of various other compounds, such as medium components, serum, bovine serum albumin, heme or metalloporphyrins [82]. Additionally, this probe, after penetrating the cell membrane, is initially deacetylated by cellular esterases to a non-fluorescent compound. This compound is then oxidized by intracellular ROS to a fluorescent 2,7-dichlorofluorescein (DCF) [80]. Therefore, when performing analyses using H_2_DCFDA, it is important to perform measurements in a medium without the addition of FBS, because this serum contains extracellular esterases that can result in high background fluorescence, which contributes to reduced sensitivity of this assay [83]. It is also worth noting that horseradish peroxidase, xanthine oxidase, catalase and superoxide dismutase also have the ability to oxidize H_2_DCFDA [80]. Moreover, cytochrome c can also significantly stimulate the formation of DCF; therefore, this should be taken into account when using this method to study oxidative stress during apoptosis, because the increase in cytosolic cytochrome c levels may overestimate fluorescence [80,84,85]. It should also be noted that DCF can be photoreduced by visible light or UVA radiation, leading to artificially increased 2,7-dichlorodihydrofluorescein oxidation and enhanced DCF fluorescence [86]. Therefore, this should be taken into account when assessing ROS formation in UVA-irradiated cells. In summary, the existence of various substances that interfere with the formation of DCF results in the fact that this method is used in cellular systems mainly as a marker of cellular oxidative stress, rather than as an indicator of the formation of H_2_O_2_ or other ROS and RNS [84]. Other authors suggest that the increased rate of DCF oxidation should be interpreted primarily as an indicator of general oxidative stress, which may be the effect of depletion of intracellular antioxidants and not necessarily evidence of increased ROS production [87]. However, despite the various limitations of this method, it is still widely used in studies of the antioxidant potential of various compounds, providing information on the level of oxidative stress in the studied cells.

### 2.3. Cytotoxicity Analysis and Cell Morphology Assessment

The next step of the study was to evaluate the effect of UVA radiation on the cytotoxicity of skin cells previously treated with *C. mas* L. extracts. For this purpose, normal human skin cells—fibroblasts (HDF) and keratinocytes (HaCaT) as well as melanoma cells (A375)—were exposed to radiation in the UVA wavelength range. The results are given as a percentage of control (cells neither treated with extracts nor exposed to UVA radiation). The effect of UVA radiation on cells not treated with extracts (+UV) was also assessed. Two tests based on different mechanisms of action were used to evaluate the cytotoxicity of the tested extracts. The Alamar Blue test was used to assess cell viability, proliferation and mitochondrial respiratory activity. On the other hand, the Neutral Red assay was used to check the integrity of cell membranes and the ability of the dye to be incorporated into lysosomes [88,89]. The results of skin cell cytotoxicity against *C. mas* L. extracts not subjected to UV radiation were published in our previous manuscript [26].

**Figure 7:**
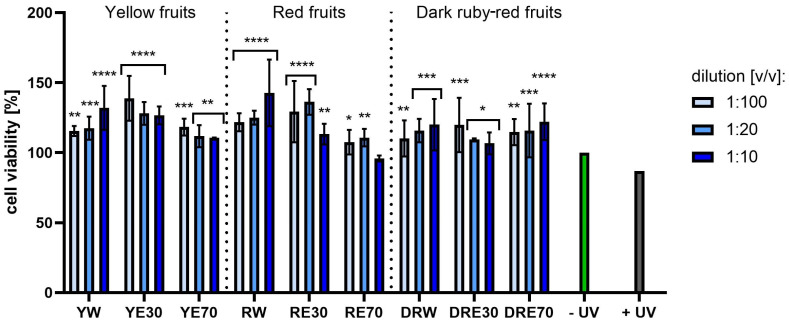
The effect of extracts from three varieties of *Cornus mas* L. (with yellow, red and dark red fruits) on the reduction of resazurin in fibroblasts after UVA radiation (5 J/cm^2^ for 1 h). Analyses were performed for water and two water–ethanolic extracts (30 and 70% (*v*/*v*)) at a dilution of 1:100, 1:20, and 1:10 (*v*/*v*). The research used yellow fruit extracts (water (YW), water–ethanol 30:70 (YE30), water–ethanol 70:30 (YE70)), red fruit extracts (water (RW), water–ethanol 30:70 (RE30), water–ethanol 70:30 (RE70)) and dark ruby-red extracts (water (DRW), water–ethanol 30:70 (DRE30), water–ethanol 70:30 (DRE70)). The exposure time to the extracts was 24 h. The positive control (+UV) were fibroblasts treated with UVA radiation (without the addition of extracts), and the negative control (−UV) were cells exposed to neither UVA radiation nor extracts, for which the viability was assumed to be 100%. Data represent mean ± SD of three independent experiments, in which each sample was tested in triplicate. **** *p* < 0.0001, *** *p* < 0.001, ** *p* < 0.01, * *p* < 0.05.

**Figure 8:**
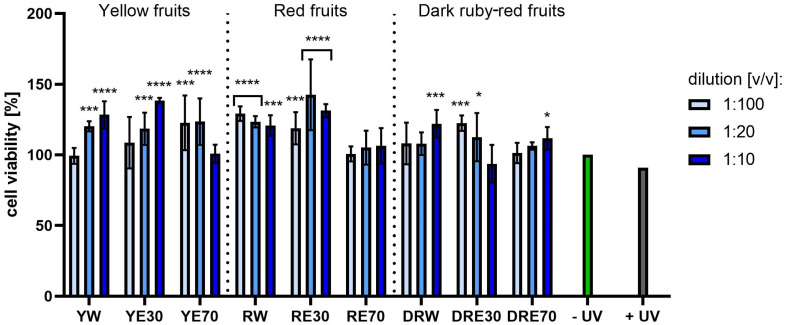
The effect of extracts from three varieties of *Cornus mas* L. (with yellow, red and dark red fruits) on the neutral red dye uptake in fibroblasts after UVA radiation (5 J/cm^2^ for 1 h). Analyses were performed for water and two water–ethanolic extracts (30 and 70% (*v*/*v*)) at a dilution of 1:100, 1:20, and 1:10 (*v*/*v*). The research used yellow fruit extracts (water (YW), water–ethanol 30:70 (YE30), water–ethanol 70:30 (YE70)), red fruit extracts (water (RW), water–ethanol 30:70 (RE30), water–ethanol 70:30 (RE70)) and dark ruby-red extracts (water (DRW), water–ethanol 30:70 (DRE30), water–ethanol 70:30 (DRE70)). The exposure time to the extracts was 24 h. The positive control (+UV) were fibroblasts treated with UVA radiation (without the addition of extracts), and the negative control (−UV) were cells exposed to neither UVA radiation nor extracts, for which the viability was assumed to be 100%. Data represent mean ± SD of three independent experiments in which each sample was tested in triplicate. **** *p* < 0.0001, *** *p* < 0.001, * *p* < 0.05.

**Figure 9:**
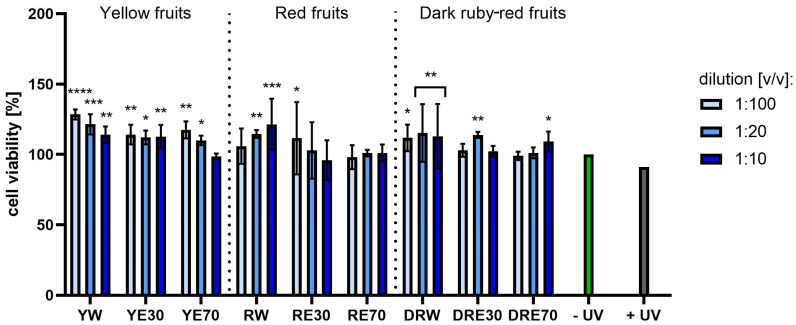
The effect of extracts from three varieties of *Cornus mas* L. (with yellow, red and dark red fruits) on the reduction of resazurin in keratinocytes after UVA radiation (5 J/cm^2^ for 1 h). Analyses were performed for water and two water–ethanolic extracts (30 and 70% (*v*/*v*)) at a dilution of 1:100, 1:20, and 1:10 (*v*/*v*). The research used yellow fruit extracts (water (YW), water–ethanol 30:70 (YE30), water–ethanol 70:30 (YE70)), red fruit extracts (water (RW), water–ethanol 30:70 (RE30), water–ethanol 70:30 (RE70)) and dark ruby-red extracts (water (DRW), water–ethanol 30:70 (DRE30), water–ethanol 70:30 (DRE70)). The exposure time to the extracts was 24 h. The positive control (+UV) were keratinocytes treated with UVA radiation (without the addition of extracts), and the negative control (−UV) were cells exposed to neither UVA radiation nor extracts, for which the viability was assumed to be 100%. Data represent mean ± SD of three independent experiments in which each sample was tested in triplicate. **** *p* < 0.0001, *** *p* < 0.001, ** *p* < 0.01, * *p* < 0.05.

**Figure 10:**
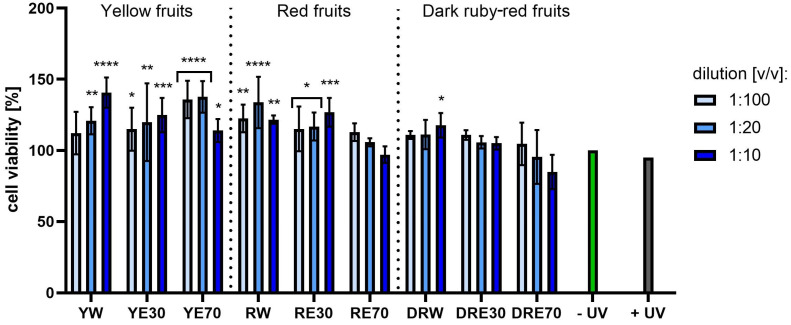
The effect of extracts from three varieties of *Cornus mas* L. (with yellow, red and dark red fruits) on the neutral red dye uptake in keratinocytes after UVA radiation (5 J/cm^2^ for 1 h). Analyses were performed for water and two water–ethanolic extracts (30 and 70% (*v*/*v* at a dilution of 1:100, 1:20, and 1:10 (*v*/*v*). The research used yellow fruit extracts (water (YW), water–ethanol 30:70 (YE30), water–ethanol 70:30 (YE70)), red fruit extracts (water (RW), water–ethanol 30:70 (RE30), water–ethanol 70:30 (RE70)) and dark ruby-red extracts (water (DRW), water–ethanol 30:70 (DRE30), water–ethanol 70:30 (DRE70)). The exposure time to the extracts was 24 h. The positive control (+UV) were keratinocytes treated with UVA radiation (without the addition of extracts), and the negative control (−UV) were cells exposed to neither UVA radiation nor extracts, for which the viability was assumed to be 100%. Data represent mean ± SD of three independent experiments, in which each sample was tested in triplicate. **** *p* < 0.0001, *** *p* < 0.001, ** *p* < 0.01, * *p* < 0.05.

**Figure 11:**
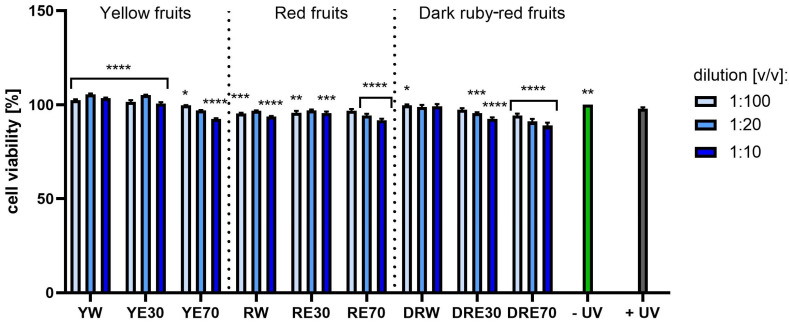
The effect of extracts from three varieties of *Cornus mas* L. (with yellow, red and dark red fruits) on the reduction of resazurin in melanoma cells after UVA radiation (5 J/cm^2^ for 1 h). Analyses were performed for water and two water–ethanolic extracts (30 and 70% (*v*/*v*)) at a dilution of 1:100, 1:20, and 1:10 (*v*/*v*). The research used yellow fruit extracts (water (YW), water–ethanol 30:70 (YE30), water–ethanol 70:30 (YE70)), red fruit extracts (water (RW), water–ethanol 30:70 (RE30), water–ethanol 70:30 (RE70)) and dark ruby-red extracts (water (DRW), water–ethanol 30:70 (DRE30), water–ethanol 70:30 (DRE70)). The exposure time to the extracts was 24 h. The positive control (+UV) were melanoma cells treated with UVA radiation (without the addition of extracts), and the negative control (−UV) were cells exposed to neither UVA radiation nor extracts, for which the viability was assumed to be 100%. Data represent mean ± SD of three independent experiments in which each sample was tested in triplicate. **** *p* < 0.0001, *** *p* < 0.001, ** *p* < 0.01, * *p* < 0.05.

**Figure 12:**
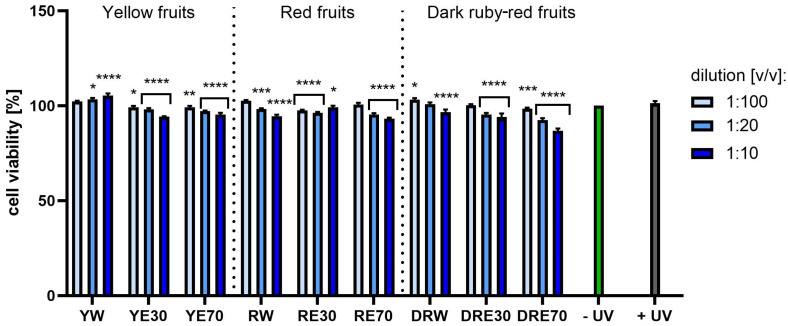
The effect of extracts from three varieties of *Cornus mas* L. (with yellow, red and dark red fruits) on the neutral red dye uptake in melanoma cells after UVA radiation (5 J/cm^2^ for 1 h). Analyses were performed for water and two water–ethanolic extracts (30 and 70% (*v*/*v*)) at a dilution of 1:100, 1:20 and 1:10 (*v*/*v*). The research used yellow fruit extracts (water (YW), water–ethanol 30:70 (YE30), water–ethanol 70:30 (YE70)), red fruit extracts (water (RW), water–ethanol 30:70 (RE30), water–ethanol 70:30 (RE70)) and dark ruby-red extracts (water (DRW), water–ethanol 30:70 (DRE30), water–ethanol 70:30 (DRE70)). The exposure time to the extracts was 24 h. The positive control (+UV) were melanoma cells treated with UVA radiation (without the addition of extracts), and the negative control (−UV) were cells exposed to neither UVA radiation nor extracts, for which the viability was assumed to be 100%. Data represent mean ± SD of three independent experiments in which each sample was tested in triplicate. **** *p* < 0.0001, *** *p* = 0.0005, ** *p* < 0.01, * *p* < 0.05.

Figure 7 and Figure 8 show the results of cytotoxicity against HDF cells after treatment with resazurin and neutral red dye. In both tests, a lack of cytotoxicity towards the cells was observed. Furthermore, a statistically significant effect of the tested extracts on HDF viability was observed, depending on the dilution used. The highest cell viability values were obtained for RW at a dilution of 1:10 (*v*/*v*), reaching 142.72 ± 23.72%; and for RE30 at a dilution of 1:20 (*v*/*v*) (142.52 ± 25.07%) for the AB and NR assays, respectively. For HaCaT cells (Figure 9 and Figure 10), the YW extract had the greatest effect on cell proliferation and metabolism, obtaining 128.44 ± 3.56% and 140.61 ± 10.63% cell viability for dilutions of 1:100 in the AB assay and 1:10 (*v*/*v*) in the NR assay, respectively. Moreover, none of the extracts at all dilutions tested showed cytotoxicity to skin cells. It is also worth mentioning that UVA radiation at the dose we used (5 J/cm^2^) (+ UV) did not significantly affect the toxicity of all cells tested, relative to unexposed cells, as also confirmed by other studies [90]. Analysing the cytotoxicity results against melanoma cells using AB and NR assays (Figure 11 and Figure 12), it was found that extracts obtained from a 70:30 mixture of alcohol and water showed the most favourable toxic effect against cells. Some extracts extracted with alcohol and water in a ratio of 30:70 also statistically reduced cell viability. In both AB and NR tests, DRE70 extract showed the most toxic effect on A375 cells, achieving 88.90 ± 1.55% and 86.90 ± 1.12% at a dilution of 1:10 (*v*/*v*) after treatment with resazurin and neutral red, respectively. What is more, for the AB test, the applied UVA dose to cells not treated with extracts statistically reduced the viability of melanoma cells.

UV radiation is divided into UVA (315–400 nm), UVB (280–315 nm) and UVC (100–280 nm). Although UVA radiation penetrates deeper than UVB and reaches the dermis, it also affects epidermal cells. The main cell type in the epidermis, the upper layer of the skin, are keratinocytes. They are equipped with a complex of non-enzymatic and enzymatic antioxidant defenses, but if excessive UVA-induced ROS production reaches critical concentrations, the normal protective components of keratinocytes are overwhelmed, which can lead to reversible or irreversible photooxidative damage to all cellular components [91]. This results in inflammation, which increases metalloproteinase activity, accelerating the degradation of collagen and elastic fibers and leading to the formation of wrinkles [92].

Therefore, many studies suggest that the use of active plant metabolites as an additive to existing photoprotective preparations is a proper choice. Plant raw materials are rich in compounds capable of absorbing UV radiation. In particular, polyphenols, are intensively studied for their UVA-protective activity [93]. The presence of double or aromatic bonds in the molecular structure of phenolic acids and flavonoids has UV absorption properties in the 200–400 nm range, making them suitable for use as sunscreen agents [94]. Many plant extracts and their active ingredients have been tested for their anti-ageing effects against various UV-induced matrix metalloproteinases in combination with antioxidant and anti-inflammatory effects in cultured skin keratinocytes and fibroblasts, as well as in mouse skin [95]. In this study, it was observed that ROS generated during UVA skin exposure increased the levels of MMPs, particularly MMP-1 in skin keratinocytes and fibroblasts responsible for the degradation of ECM proteins, including collagen and elastin, which contribute to photoaging. Plant-derived active substances also protect against melanogenesis in B16-F10 melanoma cells through stimulation of nuclear factor E2 (Nrf2) [96,97]. Despite the lack of literature data indicating a photoprotective effect of *C. mas* L. extracts, we speculate that the active compounds present in the tested extracts (Table 1) are mainly responsible for the desired effects on skin cells. In particular, the highest toxicity values in A375 cells after application of the extracts obtained with the ethanol solution may be due to the higher number of isolated flavonoids. Studies have confirmed the photoprotective effects of flavonoids on melanoma cells by scavenging ROS, regulating the cell cycle and initiating DNA repair mechanisms, inducing apoptosis and inhibiting metastasis [98,99]. The iridoids, which are so abundantly present in the tested extracts, can be responsible for the protective effect on the cells studied. A comprehensive review of iridoid derivatives highlights their significant anticancer properties by acting through induction of apoptosis, cell cycle arrest and inhibition of metastasis [100]. In addition, iridoids can influence the cell cycle of fibroblasts through modulation of key cyclin proteins and cyclin-dependent kinases (CDKs). Iridoids can stimulate fibroblast migration to the site of injury and promote tissue repair processes by increasing ECM protein production and angiogenesis [101,102]. Furthermore, the iridoid compound, geniposide, has been shown to protect fibroblasts cells from UVB-induced apoptosis through antioxidant mechanisms and inhibition of apoptotic pathways. It acts by neutralizing ROS, which prevents DNA and protein damage, and reduces the activation of caspases, which are key enzymes in the apoptosis process [103]. In summary, the polyphenols present in the studied extracts act mainly by scavenging free radicals, modulating signaling pathways and inhibiting inflammatory responses. These mechanisms include reducing ROS, decreasing the expression of pro-inflammatory cytokines and increasing the activity of antioxidant enzymes [104,105,106]. In addition, other studies have shown that extracts from the yellow and red fruits of *C. mas* L. induced cytotoxicity in A375 and MeWo melanoma cells, as well as other cancer cell lines [107,108,109,110].

Additionally, as a part of this work, photos of the tested cells (HDF, HaCaT and A375) were taken using an inverted microscope to assess their morphology after exposure to both UVA radiation and the tested extracts. The analyses showed that neither UVA nor the tested dogwood fruit extracts significantly affected the morphology of normal cells. However, in the case of melanoma cells, the use of ethanol extracts (70% (*v*/*v*)) significantly affects the cells, inhibiting their adhesion to the bottom of the cell culture dishes (Figures S2–S4).

### 2.4. Assessment of Melanin Formation and Tyrosinase Activity Inhibition

**Figure 13:**
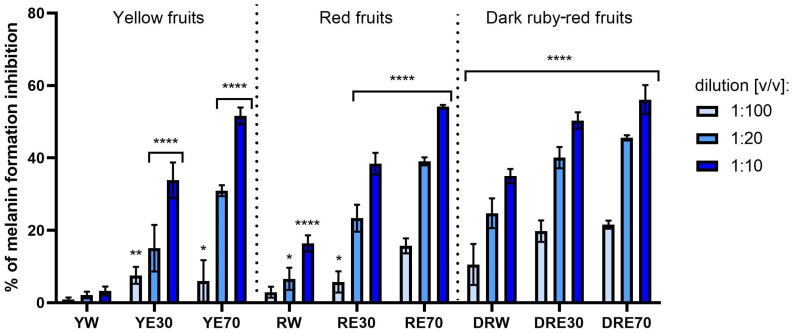
Assessment of inhibition of melanin formation by water, ethanol–water (30:70 (*v*/*v*)) and ethanol–water (70:30 (*v*/*v*)) extracts from three varieties of *C. mas* L. Extracts were obtained from varieties with yellow, red and dark ruby-red fruit. Analyses were performed for extracts at dilution of 1:100, 1:20 and 1:10 (*v*/*v*). The research used yellow fruit extracts (water (YW), water–ethanol 30:70 (YE30), water–ethanol 70:30 (YE70)), red fruit extracts (water (RW), water–ethanol 30:70 (RE30), water–ethanol 70:30 (RE70)) and dark ruby-red extracts (water (DRW), water–ethanol 30:70 (DRE30), water–ethanol 70:30 (DRE70)). Data represent mean ± SD of three independent experiments in which each dilution of individual dogwood extracts was tested in triplicate. **** *p* < 0.0001, ** *p* = 0.0029, * *p* < 0.05.

**Figure 14:**
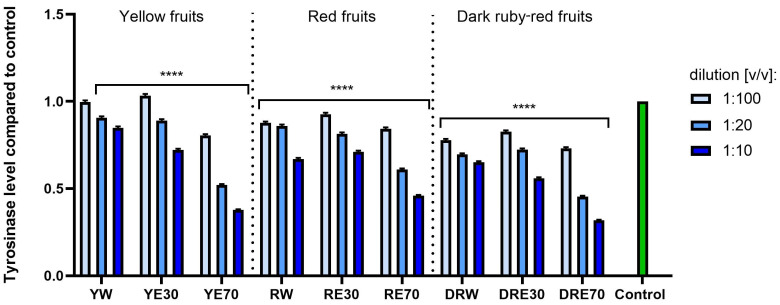
Assessment of tyrosinase level in B16F10 melanoma cells after exposure to water, ethanol–water (30:70 (*v*/*v*)) and ethanol–water (70:30 (*v*/*v*)) extracts from three varieties of *C. mas* L. Extracts were obtained from varieties with yellow, red and dark ruby-red fruit. Analyses were performed for extracts at dilutions of 1:100, 1:20 and 1:10 (*v*/*v*). The research used yellow fruit extracts (water (YW), water–ethanol 30:70 (YE30), water–ethanol 70:30 (YE70)), red fruit extracts (water (RW), water–ethanol 30:70 (RE30), water–ethanol 70:30 (RE70)) and dark ruby-red extracts (water (DRW), water–ethanol 30:70 (DRE30), water–ethanol 70:30 (DRE70)). Data represent mean ± SD of three independent experiments in which each dilution of individual extracts was tested in triplicate. **** *p* < 0.0001.

Due to the fact that excessive exposure to UV radiation is often associated with the formation of numerous discolorations on the skin surface, the aim of this study was also to assess the possibility of inhibiting melanin formation. This chemical compound is produced by melanocytes during the process of melanogenesis, catalyzed by a number of different proteins [111]. Excessive exposure of the skin to UV radiation causes photoaging accompanied by uneven distribution of the pigment melanin. These discolorations appear primarily when melanin is produced in excess and its distribution on the skin surface is disturbed. This leads to the formation of unsightly spots, the number of which increases with age [2]. UV radiation also induces the production of ROS in cells, which also stimulates the process of melanogenesis, thus increasing the amount of skin discoloration [78]. Therefore, as a part of this work, it was assessed whether the obtained dogwood extracts can affect the formation of melanin, contributing to inhibition of the process of pigmentation disorders. The analyses performed indicated inhibition of melanin formation, depending on the type of extract and the dilution used (Figure 13). The synthesis of this pigment was inhibited most strongly by extracts from dark red fruits, slightly less by red fruit and the least by yellow fruit. The solvent used during the extraction process played an important role in inhibiting the production of this pigment. The strongest inhibition was observed in the case of extracts obtained using a mixture of ethanol and water in the ratio 70:30 (*v*/*v*). In the case of these extracts at the lowest tested dilution of 1:10 (*v*/*v*), inhibition of 56.11% for DRE70, 54.23% for RE70 and 51.63% for YE70 was obtained. The cellular tyrosinase activity assay results indicated that the activity of tyrosinase in murine melanoma B16F10 cells decreases in all the extracts tested. With increased concentration of the extracts, the activity of this enzyme is lower (Figure 14). Similarly to the inhibition of melanin formation, the extracts obtained using 70% ethanol solution have a stronger effect on the tyrosinase activity, for which at a dilution of 1:10 (*v*/*v*), the tyrosinase activity decreases more than 3-fold. So far, the literature data have not indicated the ability to inhibit tyrosinase activity by extracts from various varieties of *C. mas* L. The available data indicate various mechanisms of inhibition of melanin formation by plant-derived compounds [112]. This effect is most often attributed to the possibility of inhibition of tyrosinase activity by phytochemicals present in plant extracts [112,113,114]. Another possible mechanism is the downregulation of microphthalmia-associated transcription factors (MITF) gene expression by affecting various signaling pathways and mediating cytokine autophagy [112,115,116]. The antimelanogenic effect may also be related to the absorption of UV radiation by compounds present in plant extracts, such as flavonoids [112,117]. Although there are no literature reports on the inhibition of tyrosinase activity by dogwood extracts, this is one of the possible mechanisms responsible for the inhibition of melanin formation observed in this work. The literature data only indicate a strong anti-tyrosinase activity of iridoid fractions isolated from the fruits of this plant, which have the ability to inhibit this enzyme by 88.9% [106]. These authors also point to a stronger activity of iridoids isolated from water–ethanol extracts compared to water extracts [118]. The highest anti-melanogenic activity of our extracts obtained using ethanol solution may be due to the larger number of isolated flavonoids. These compounds have proven ability to inhibit the activity of this enzyme and control the production of melanin [119]. According to the literature data, the activity of these phytochemicals is strictly dependent on their structure, and the chelation of copper atoms in the tyrosinase molecule may be the effect of the polyhydroxylated phenolic structure of this group of compounds [119,120]. Isoprene moieties and flavonoid dimerization also play an important role [121,122]. Jakimiuk et al. showed that the flavonoid core with the hydroxyl group at the C7 position is important in the antityrosinase activity of these compounds. Hydroxylation at C3 and methylation at C8, C7 and C3 in their benzo-γ-pyran ring also have a significant impact on inhibiting the activity of this enzyme [123]. Moreover, the literature data indicate that some compounds belonging to this group inhibit the expression of this enzyme by modulating certain signaling pathways, such as phosphorylation of MITF, CREB, AMPK and MAPK proteins, as well as p38 [124,125,126]. Although the exact mechanism of anti-melanogenic action of the tested extracts requires further analysis, the ability to inhibit melanin formation shown in this work indicates the potential of dogwood fruit in protecting against skin discoloration. The ability of dogwood to inhibit melanin production by fungal tyrosinase and to reduce tyrosinase activity in a mouse model of melanoma indicate the validity of further studies on this plant in the context of skin pigmentation disorders.

## 3. Methods and Materials

### 3.1. Reagents

In our study, 2,2-Azino-bis-3-ethylbenzothiazoline-6-sulfonic acid (ABTS solution), 2,2-diphenyl-1-picrylhydrazyl (DPPH), 2,4,6-Tris(2-pyridyl)-s-triazine (TPTZ), acetonitrile, ascorbic acid (vitamin C), L-DOPA, kojic acid, MS–grade formic acid, neutral red solution (NR), phenylmethylsulfonyl fluoride (PMSF), resazurin sodium salt (RES), Triton X-100, Trolox, trypsin-EDTA solution and tyrosinase from mushroom were purchased from Merck KGaA (Darmstadt, Germany). Acetic acid (CH_3_COOH), ethanol (96%), iron (III) chloride hexahydrate (FeCl_3_ × 6H_2_O), methanol, potassium persulfate and sodium acetate (CH_3_COONa) were acquired from Warchem (Zakręt, Poland). DMEM (Dulbecco’s Modification of Eagle’s Medium), FBS (Fetal Bovine Serum) and PBS (phosphate buffered saline, pH 7.00 ± 0.05) were purchased from VWR International (Radnor, PA, USA). 2′,7′-dichlorodihydrofluorescein diacetate (H_2_DCFDA) and antibiotics (penicillin-streptomycin) were acquired from Thermo Fisher Scientific (Waltham, MA, USA).

### 3.2. Plant Material and Extraction Procedure

For the analyses, three varieties of *C. mas* L. fruits were obtained from a local producer: Jantarnyj (yellow fruit), Korralowyj Marka (red fruit), and Bolestraszycki (dark ruby-red fruit). The deposit numbers of the fruit used are CMJ260823, CMKM280823 and CMB020923, respectively. The study used ultrasound-assisted extraction according to Yang et al. with minor modifications [127]. Three types of extracts were prepared for each variety: water (5 g of fruit and 100 mL of distilled water), water–ethanol in a 70:30 ratio (5 g of fruit, 70 mL of distilled water, 30 mL of ethanol), and water–ethanol in a 30:70 ratio (5 g of fruit, 30 mL of distilled water, 70 mL of ethanol). The extraction process was carried out on a magnetic stirrer (Chemland, Stargard, Poland) for 14 h, and then the extracts were sonicated in an ultrasonic bath (Digital Ultrasonic Cleaner, Cincinnati, OH, USA) for 30 min. Ethanol was gently evaporated, water was added to a final volume of 100 mL, and the extracts were sonicated for 15 minutes. The extracts obtained from different varieties of *C. mas* L. fruit were filtered using Whatman filter paper No. 10 (Thermo Fisher Scientific, Waltham, MA, USA). The extracts prepared in this way (not evaporated to dryness) were diluted 10-fold, 20-fold and 100-fold, respectively. In the DPPH, FRAP and melanin formation inhibition tests, dilutions of the obtained extracts were performed in distilled water. In the ABTS assay, dilutions were made in PBS solution, and in all cell culture tests, DMEM medium was used. During the analyses, the extracts were stored at approximately 4 °C. After the analyses, the extracts were stored at − 80°C.

### 3.3. Determination of Biologically Active Compounds

Reference compounds and reagents (MS–grade formic acid and acetonitrile) were purchased from Sigma–Aldrich (St. Louis, MO, USA). Ultrapure Millipore Direct-Q^®^ 3UV–R system (Merck, KGaA, Germany) was used to obtain deionized water.

UHPLC apparatus (Infinity Series II) with a DAD detector and an Agilent 6224 ESI/TOF mass detector was from Agilent Technologies (Santa Clara, CA, USA). Separation was carried out on Titan RP18 column (Supelco, Sigma–Aldrich, Burlington, MA, USA) (10 cm × 2.1 mm i.d. and a particle size of 1.9 µm). The chromatographic conditions and mass spectrometry parameters were described in detail previously [128].

### 3.4. Determination of Antioxidant Properties

#### 3.4.1. ABTS Scavenging Assay

To evaluate the antioxidant capacity of extracts obtained from different *C. mas* L. varieties, the ABTS Scavenging Assay described by Miller et al. [129] was performed. Initially, a 7 mM ABTS solution (Merck KGaA, Darmstadt, Germany) and a 2.4 mM potassium persulfate solution (Merck KGaA, Darmstadt, Germany) were mixed in equal proportions and left for 14 h. After 14 h, the prepared solution was diluted with PBS (VWR International, Radnor, PA, USA) to obtain an absorbance of 1 ± 0.04 at λ = 734 nm. Then, analyses of dogwood extracts were performed by mixing the ABTS solution and the tested samples. Trolox and ascorbic acid (Merck KGaA, Darmstadt, Germany) were used as positive controls, PBS as a negative control, and the extract without ABTS as a blank. Absorbances were measured at 734 nm using a UV/VIS spectrophotometer (Thermo Fisher Scientific, Waltham, MA, USA). To ensure that the absorbance of the samples by themselves is not influencing the results, absorption spectra of extracts were performed at 1:10 and 1:100 (*v*/*v*) dilutions using DR 600 spectrophotometer (Hach Lange, Wrocław, Poland) (Figure S5). The analyses were performed twice for each dilution and the entire experiment was repeated three times. Using Equation (1), the percentage of ABTS free radical scavenging was calculated.

(1) $$\%\;of\;ABTS\;scavenging=1-\frac{Abs\;sample}{Abs\;control}\times 100$$

where: Abs sample—absorbance of the sample; Abs control—absorbance of the control sample (mixture of PBS and ABTS).

#### 3.4.2. DPPH (1,1-Diphenyl-2-picrylhydrazyl) Radical Scavenging Assay

To evaluate the antioxidant properties of the studied *C. mas* L. fruit extracts, the DPPH radical assay was used, as described by Brand-Williams et al. [130]. For this purpose, the extracts obtained were diluted in distilled water to obtain dilutions of 1:10, 1:20 and 1:100 (*v*/*v*). Then, 100 μL of each sample (in triplicate) was placed in wells of a 96-well plate. Then, 100 μL of a 4 mM methanol solution of DPPH (Merck KGaA, Darmstadt, Germany) was added to each well and thoroughly mixed. Distilled water was used as a negative control instead of the test sample. The plate was placed in a plate reader, and absorbance at λ = 517 nm was measured every 5 min for 20 min. Analyses were performed in triplicate for each dilution, and the entire experiment was repeated three times. The percentage of DPPH free radical scavenging was calculated using Equation (2).

(2) $$\%\;DPPH\;scavenging=\frac{Abs\;control-Abs\;sample}{Abs\;control}\times 100$$

where: Abs sample—absorbance of the sample; Abs control—absorbance of the control sample.

#### 3.4.3. Determination of Ferric Reducing Antioxidant Power (FRAP Assay)

The antioxidant capacity was determined using the spectrophotometric FRAP assay, conducted according to Benzie and Strain [131]. A FRAP mixture was prepared, consisting of 0.3 M acetate buffer, 0.01 M TPTZ (Merck KGaA, Darmstadt, Germany), and 0.02 M FeCl_3_ × 6H_2_O in proportions of 10:1:1. Then, 180 µL of the FRAP mixture and 20 µL of the tested samples were added to a 96-well plate. The FRAP mixture and distilled water were used as a blank. The plates were incubated for 20 min, and the absorbance was measured at λ = 593 nm. A standard curve was prepared using Trolox (Merck KGaA, Darmstadt, Germany) by adding 180 µL of the FRAP mixture and 20 µL of Trolox at appropriate concentrations (0–800 µM). The entire experiment was repeated three times, with each sample analyzed in triplicate. The results were calculated based on the calibration curve formula and expressed in Trolox equivalents (µmol TE/L).

#### 3.4.4. Determination of Intracellular Levels of Reactive Oxygen Species (ROS)

To evaluate the ability of fruit extracts from three different varieties of *C. mas* L. to reduce the intracellular production of reactive oxygen species in fibroblasts, keratinocytes (HaCaT) and human melanoma cells (A375), the fluorogenic dye 2′,7′-dichlorodihydrofluorescein diacetate (H_2_DCFDA) (Thermo Fisher Scientific, Waltham, MA, USA) was used. Cells were seeded in 96-well plates and incubated for 24 h. Then the medium was replaced with solutions of individual extracts dissolved in DMEM (VWR International, Radnor, PA, USA) at dilutions of 1:10, 1:20 and 1:100 (*v*/*v*) and incubated for another 24 h. After that time, the DMEM was aspirated and replaced with PBS. Then the cells were exposed to UVA radiation at a dose of 5 J/cm^2^ for 1 h. Cells exposed to UVA irradiation but not to the tested extracts were used as positive controls. Cells exposed neither to UVA nor to the tested extracts were used as negative controls. After UVA irradiation of cells, the solutions were aspirated from the wells and replaced with 200 µL of 10 µM H_2_DCFDA indicator solution dissolved in serum-free DMEM. Measurements were performed at an excitation wavelength of λ = 485 nm and an emission wavelength of λ = 530 nm using a microplate reader (FilterMax F5, Thermo Fisher Scientific, Waltham, MA, USA). The analysis was repeated three times, in which each sample was analyzed in triplicate.

### 3.5. Cytotoxicity Analysis

#### 3.5.1. Cell Culture

Three cell lines were used for cytotoxicity analyses, including two normal human skin cell lines (fibroblasts (HDF) and keratinocytes (HaCaT)) and human melanoma cells (A375). Normal cells were purchased from CLS (Cell Lines Service in Eppelheim, Eppelheim, Germany) and human melanoma cells were obtained from ATCC (American Type Culture Collection Manassas, VA, USA). Additionally, murine melanoma B16F10 cells, also obtained from ATTC, were used to assess cellular tyrosinase activity. The first three cell lines were cultured in culture flasks using DMEM (Dulbecco’s Modified Eagle Medium from VWR International, Radnor, PA, USA) with high glucose content, supplemented with 1% (*v*/*v*) antibiotics (100 U/mL penicillin and 1000 μg/mL streptomycin from Thermo Fisher Scientific, Waltham, MA, USA) and 10% (*v*/*v*) fetal bovine serum (FBS from VWR International, Radnor, PA, USA). RPMI 1640 medium (Merck KGaA, Darmstadt, Germany) supplemented analogously to the DMEM medium was used to culture B16F10 cells. When the cells reached an appropriate confluence (approximately 70–80%), the cells were rinsed twice with sterile PBS (VWR International, Radnor, PA, USA) and were trypsinized to detach them from the culture flasks. Cells were then seeded into 96-well plates at a density of 1 × 10^4^ cells/well and incubated for 24 h to prepare cells for cytotoxicity assays.

#### 3.5.2. Alamar Blue Assay

The first test performed to assess skin cell viability was the Alamar Blue Assay. This test was performed according to the procedure described by Page et al. [132] and Gag et al. [90] with modifications. Briefly, after 24 h of cell culture, the cells were exposed to nine dogwood extracts for 24 h. After this time, the DMEM with the tested extracts was aspirated, replaced with sterile PBS, and the cells were then exposed to UVA radiation (λ = 366 nm) at a dose of 5 J/cm^2^ for 1 h using a UV analysis lamp (KRÜSS OPTRONIC GMBH, Hamburg, Germany). The positive control (+UV) consisted of fibroblasts exposed to UVA radiation (without the addition of extracts), and the negative control (−UV) consisted of cells not exposed to either UVA radiation or extracts. After 24 h, the DMEM were aspirated and 200 μL of a 6 mM resazurin solution (Merck KGaA, Darmstadt, Germany) was added to each well. The plates were then incubated for 2 h at 37 °C. After this time, fluorescence was measured at λ = 570 nm using a FiterMax F5 microplate reader (Thermo Fisher Scientific, Waltham, MA, USA). The entire study was repeated three times, and each sample was analyzed three times. Results are presented as percentage of negative control (−UV).

#### 3.5.3. Neutral Red Uptake Assay

The neutral red assay was the second test performed to assess the viability of skin cells, which was carried out according to the procedure described by Borenfreund et al. [133] and Gag et al. [90], with modifications. Similar to the Alamar Blue Assay, after a 24 h of incubation of the cells with the tested extracts, the DMEM was aspirated, replaced with sterile PBS, and the cells were exposed to UVA radiation (λ = 366 nm) at a dose of 5 J/cm^2^ for 1 h using UV analysis lamp (KRÜSS OPTRONIC GMBH, Hamburg, Germany). The positive control (+UV) were fibroblasts exposed to UVA radiation (without the addition of extracts), and the negative control (−UV) were cells not exposed to either UVA radiation or extracts. After 24 h, the DMEM medium was aspirated and 100 μL of a 40 μg/mL neutral red dye solution (Merck KGaA, Darmstadt, Germany) was applied. Following a 2 h incubation, the NR dye solution was removed. The cells were rinsed with sterile PBS, which was then aspirated. Subsequently, 150 µL of decolorizing buffer, consisting of C_2_H_5_OH, CH_3_COOH and H_2_O in a ratio of 50%/1%/49%, was added to each well. Absorbance was measured using a microplate reader at λ = 570 nm (Thermo Fisher Scientific, Waltham, MA, USA). The entire experiment was repeated three times, with each sample analyzed in triplicate. The results were presented as a percentage of the negative control (−UV).

#### 3.5.4. Cell Morphology Assessment

In order to assess the morphology of cells exposed to UVA radiation (5 J/cm^2^ for 1 h) and the tested extracts (24 h, at a dilution of 1:10 (*v*/*v*)), microscopic images of fibroblasts (HDF), keratinocytes (HaCaT) and melanoma cells (A375) were taken using an inverted microscope (Nikon EclipseTS 100-F; Nikon, Warsaw, Poland) at 10× magnification (scale bar: 100 μm). Cells were cultured in 35 mm diameter cell culture dishes.

### 3.6. Evaluation of Inhibition of Melanin Formation

The assessment of the ability to inhibit melanin formation by the tested extracts from *C. mas* L. was carried out according to the procedure previously described by Krochmal-Marczak et al. [134]. For this purpose, 20 µL of all types of extracts (at dilutions of 1:10, 1:20 and 1:100 (*v*/*v*)) were added to individual wells in a 96-well plate. In the next step, fungal tyrosinase solution (500 U/mL) and phosphate buffer (pH 6.8) were added to the wells. The plates were then thoroughly mixed in the dark on an orbital shaker for 10 min at 25 °C. L-DOPA (Merck KGaA, Darmstadt, Germany) was then added to the wells as a substrate and incubated for 20 min in the dark. Kojic acid at a concentration of 500 µg/mL was used as a positive control. Spectrophotometric measurements were then made at λ = 475 nm using a microplate reader (FilterMax F5, ThermoFisher Scientific, Waltham, MA, USA). The assessment of the inhibition of melanin production was performed in triplicate for each dilution of the tested extracts. The inhibitory effects of the tested extracts were expressed as a percentage of melanin formation inhibition, as follows:

(3) $$\%\;melanin\;formation\;inhibition=\frac{\left(A-B\right)-(C-D)}{A-B}\times 100$$

where: A—optical density of the mixture without a test sample; B—optical density of the mixture without a test sample an enzyme; C—optical density of the mixture with a test sample and an enzyme; D—optical density of the mixture without an enzyme.

### 3.7. Evaluation of Cellular Tyrosinase Activity in Melanoma Cells

Tyrosinase activity in murine melanoma B16F10 cells exposed to the tested *C. mas* L. extracts was assessed using a slightly modified methodology described by Akaberi et al. [135]. For this purpose, B16F10 cells were exposed to the analyzed extracts (at dilutions of 1:100, 1:20 and 1:10 (*v*/*v*)) for 24 h. The extracts were then aspirated, replaced with sterile PBS and exposed to UVA radiation (λ = 366 nm) at a dose of 5 J/cm^2^ for 1 h using a UV analysis lamp (KRÜSS OPTRONIC GMBH, Hamburg, Germany). The control cells were B16F10 cells not exposed to the tested extracts. Then, PBS was aspirated and cells were trypsinized. The collected cells were pelleted and washed with PBS. Then, pelleted cells were lysed with 100 µL of 100 mM sodium phosphate buffer (pH 6.8) containing 1% Triton X-100 and 0.1 mM protease inhibitor (PMSF). After 30 min, cell lysates were centrifuged at 10,000 rpm for 20 min at 4 °C. The obtained supernatants were transferred to sterile microtubes and stored at −80 °C for 30 min. In the next step, 100 µL of the obtained protein suspension and 100 µL of DOPA (5 mM) were added to the wells of a 96-well plate. The plates were incubated for 2 h, after which the absorbance at 475 nm was measured. Three independent experiments were performed, in which each tested sample was performed in triplicate.

### 3.8. Statistical Analysis

Statistical analysis of the obtained results was performed using the GraphPad Prism 8.4.3 program (GraphPadSoftware, Inc., San Diego, CA, USA). The values obtained during the analyses performed were presented as means ± standard deviations (SD). As a normal distribution was confirmed, analysis of variance (ANOVA) was performed. The significance of differences in the content of biologically active compounds between individual dogwood extracts was assessed using the Tukey test (α < 0.05). Statistically significant differences between the content of individual compounds in the extracts tested were marked with different letters. In order to assess differences in the biological activity of the tested extracts compared to the control were assessed using Dunnett’s post-test (α < 0.05). The statistical significance of the obtained values was determined at **** *p* < 0.0001, *** *p* < 0.001, ** *p* < 0.01 and * *p* < 0.05 compared to the control group.

## 4. Conclusions

The results obtained in this work confirmed that extracts from dogwood fruit could be a valuable cosmetic raw material with multidirectional effect. Water–ethanol extracts from yellow and dark ruby-red fruits seem to be particularly promising. Although many properties of this plant have been proven so far, this work indicates its anti-melanogenic and photoprotective potential. It therefore seems justified to conduct further studies focusing on understanding the exact mechanisms of action of these extracts in the context of inhibiting melanin production and protecting the skin from the effects of various types of UV radiation. Moreover, this work demonstrated the ability to reduce the viability of melanoma cells by *C. mas* L. fruit extracts, which indicates the validity of further studies on their anti-cancer potential in the context of skin cancers.

## Figures and Tables

**Table 1:** Concentration of the components (µg/mL ± SD) found in extracts prepared from *C. mas* L. fruit.

Compound	YW	YE30	YE70	RW	RE30	RE70	DRW	DRE30	DRE70
**Gallic acid and derivatives**									
Galloyl hexoside	12.22 ± 0.57 ^a^	7.81 ± 0.40 ^b^	6.25 ± 0.55 ^c^	2.70 ± 0.09 ^d^	1.79 ± 0.05 ^e^	0.99 ± 0.08 ^f^	7.94 ± 0.48 ^b^	4.69 ± 0.60 ^c^	1.56 ± 0.07 ^e^
Gallic acid *	1.65 ± 0.11 ^b^	2.11 ± 0.11 ^a^	0.99 ± 0.08 ^c^	1.04 ± 0.09 ^c^	ND	ND	1.48 ± 0.13 ^b^	ND	ND
Galloyl-d-sedoheptulose	11.32 ± 1.19 ^b^	6.98 ± 0.40 ^c^	4.34 ± 0.41 ^d^	5.42 ± 0.62 ^d^	4.34 ± 0.48 ^d^	2.51 ± 0.12 ^e^	13.99 ± 0.98 ^a^	10.08 ± 1.12 ^b^	3.57 ± 0.20 ^d^
**Iridoids**									
Loganic acid *	163.9 ± 12.8 ^c^	278.0 ± 13.4 ^a^	196.4 ± 10.5 ^b^	69.25 ± 3.25 ^e^	81.83 ± 4.94 ^e^	77.23 ± 3.58 ^e^	130.6 ± 11.5 ^d^	151.4 ± 9.1 ^c^	102.1 ± 3.5 ^f^
Cornuside *	3.66 ± 0.18 ^b^	7.65 ± 0.41 ^a^	7.32 ± 0.41 ^a^	1.50 ± 0.11 ^e^	1.77 ± 0.08 ^b^	2.88 ± 0.19 ^c^	2.29 ± 0.19 ^c,d^	2.13 ± 0.13 ^d^	2.55 ± 0.14 ^c^
**Cyclohexanecarboxylic and phenolic acids**									
Quinic acid *	51.81 ± 2.10 ^a^	54.33 ± 1.90 ^a^	53.71 ± 2.34 ^a^	38.18 ± 1.53 ^b^	36.53 ± 2.09 ^b^	35.76 ± 2.50 ^b^	50.20 ± 2.16 ^a^	51.08 ± 2.01 ^a^	50.65 ±1.90 ^a^
Protocatechuic acid *	0.09 ± 0.01 ^b^	ND	ND	0.10 ± 0.01 ^b^	0.07 ± 0.01 ^c^	0.05 ± 0.01 ^c^	0.18 ± 0.02 ^a^	0.20 ± 0.02 ^a^	0.13 ± 0.01 ^b^
Caftaric acids *	2.32 ± 0.16 ^b^	3.05 ± 0.21 ^a^	2.87 ± 0.22 ^a^	1.55 ± 0.14 ^d^	1.60 ± 0.09 ^c,d^	1.68 ± 0.11 ^c,d^	1.72 ± 0.13 ^c^	1.84 ± 0.12 ^c^	1.66 ± 0.14 ^c,d^
Chlorogenic acid *	0.90 ± 0.04 ^c^	1.50 ± 0.10 ^a^	1.14 ± 0.06 ^b^	0.49 ± 0.02 ^d^	0.56 ± 0.03 ^d^	0.64 ± 0.04 ^d^	0.82 ± 0.07 ^c^	1.13 ± 0.16 ^b^	0.86 ± 0.04 ^c^
coumaroylquinic acids	0.39 ± 0.02 ^c^	0.81 ± 0.06 ^b^	0.82 ± 0.07 ^b^	0.29 ± 0.02 ^d^	0.45 ± 0.05 ^c^	0.85 ± 0.06 ^b^	0.44 ± 0.05 ^c^	1.02 ± 0.10 ^a^	1.01 ± 0.11 ^a^
p-coumaric acid	0.31 ± 0.01 ^d^	0.66 ± 0.03 ^b^	0.63 ± 0.04 ^b^	0.27 ± 0.02 ^d^	0.30 ± 0.02 ^b^	0.36 ± 0.02 ^c,d^	0.40 ± 0.02 ^c^	0.82 ± 0.04 ^a^	0.79 ± 0.03 ^a^
Hydroxybenzoic glucoside	0.42 ± 0.02 ^c^	0.82 ± 0.04 ^b^	1.04 ± 0.11 ^a^	0.39 ± 0.04 ^c^	0.25 ± 0.03 ^d^	0.06 ± 0.00 ^f^	0.80 ± 0.04 ^b^	1.01 ± 0.09 ^a^	0.82 ± 0.09 ^b^
**Anthocyanins**									
Cyanidin 3-*O*-galactoside *	ND	ND	ND	0.71 ± 0.07 ^f^	1.48 ± 0.10 ^e^	2.03 ± 0.18 ^d^	3.97 ± 0.22 ^c^	10.62 ± 0.97 ^a^	7.64 ± 0.54 ^b^
Pelargonidin 3-*O*-glucoside *	ND	ND	ND	1.21 ± 0.08 ^f^	3.74 ± 0.23 ^d^	4.21 ± 0.41 ^d^	6.67 ± 0.51 ^c^	19.14 ± 0.81 ^a^	11.07 ± 0.91 ^b^
**Flavonoids**									
Quercetin 3-glucuronide *	2.32 ± 0.19 ^e^	9.24 ± 0.45 ^b^	16.91 ± 1.37 ^a^	1.87 ± 0.17 ^e^	2.22 ± 0.19 ^e^	6.35 ± 0.55 ^c^	3.28 ± 0.31 ^d^	7.42 ± 0.66 ^c^	14.26 ± 0.99 ^a^
Kaempferol 3-*O*-galactoside	ND	ND	ND	0.89 ± 0.06 ^c^	0.58 ± 0.05 ^d^	1.06 ± 0.09 ^c^	3.12 ± 0.14 ^b^	4.77 ± 0.21 ^a^	2.96 ± 0.13 ^b^

* Identification was confirmed using standard: ND—not detected; YW—yellow-fruit water extract; YE30 and YE70—yellow-fruit extract obtained using 30% and 70% ethanol, respectively; RW—red-fruit water extract; RE30 and RE70—red-fruit extract obtained using 30% and 70% ethanol, respectively; DRW—dark ruby-red water extract; DRE30 and DRE70—dark ruby-red extract obtained using 30% and 70% ethanol, respectively. The presence of different letters on the same lines indicates a statistically significant difference (*p* < 0.05).

## Data Availability

The data presented in this study are available on request from the corresponding author.

## Supplementary Materials

The following supporting information can be downloaded at: https://www.mdpi.com/article/10.3390/ijms252010993/s1, Table S1. Data used for identification of components the extracts from *C. mas* L. fruits; Figure S1. Representative chromatograms obtained for 70% ethanol extracts from red-fruit (blue line), yellow-fruit (green line) and dark ruby-red fruit (red line) of *C. mas* L. varieties. a- chromatograms recorded in negative ionization mode (BPC chromatogram); b—DAD chromatograms recorded at λ = 254 nm; c—DAD chromatograms recorded at λ = 320 nm.
