# Supplementary material for: Assessment of the Antioxidant and Photoprotective Properties of Cornus mas L. Extracts on HDF, HaCaT and A375 Cells Exposed to UVA Radiation
Source: Int J Mol Sci. 2024 Oct 12;25(20):10993. doi: 10.3390/ijms252010993 (PMC11507244; doi:10.3390/ijms252010993)
Supplement: Supplementary file 1 [file ijms-25-10993-s001.zip › ijms-3217560-supplementary.pdf]

International Journal of *Molecular Sciences*

Article

Assessment of the Antioxidant and Photoprotective Properties of *Cornus mas* L. Extracts on HDF, HaCaT and A375 Cells Exposed to UVA Radiation

Martyna Zagórska-Dziok [1], Agnieszka Mokrzyńska [1], Aleksandra Ziemlewska [1], Zofia Nizioł-Łukaszewska [1], Ireneusz Sowa [2], Marcin Feldo [3] and Magdalena Wójciak [2,*]

[1] Department of Technology of Cosmetic and Pharmaceutical Products, Medical College, University of Information Technology and Management in Rzeszow, Sucharskiego 2, 35-225 Rzeszow, Poland; mzagorska@wsiz.edu.pl (M.Z.-D.); amokrzynska@wsiz.edu.pl (A.M.); aziemlewska@wsiz.edu.pl (A.Z.); zniziol@wsiz.edu.pl (Z.N.-Ł.)

[2] Department of Analytical Chemistry, Medical University of Lublin, Aleje Raclawickie 1, 20-059 Lublin, Poland; i.sowa@umlub.pl

[3] Department of Vascular Surgery, Medical University of Lublin, Staszica 11 St., 20-081 Lublin, Poland; martinf@interia.pl

* Correspondence: magdalena.wojciak@umlub.pl

Citation: Zagórska-Dziok, M.; Mokrzyńska, A.; Ziemlewska, A.; Nizioł-Łukaszewska, Z.; Sowa, I.; Feldo, M.; Wójciak, M. Assessment of the Antioxidant and Photoprotective Properties of *Cornus mas* L. Extracts on HDF, HaCaT and A375 Cells Exposed to UVA Radiation. *Int. J. Mol. Sci.* **2024**, *25*, 10993. https://doi.org/10.3390/ijms252010993

Academic Editor: Cheng-Yang Huang

Received: 4 September 2024
Revised: 9 October 2024
Accepted: 10 October 2024
Published: 12 October 2024

Copyright: © 2024 by the authors. Licensee MDPI, Basel, Switzerland. This article is an open access article distributed under the terms and conditions of the Creative Commons Attribution (CC BY) license (https://creativecommons.org/licenses/by/4.0/).

Table S1. Data used for identification of components the extracts from *C. mas* L. fruits.

Rt (min)	Observed ion mass [M-H]-	Δ ppm	Formula	Identified
1.6	191.05600	-0.58	$C_7H_{12}O_6$	Quinic acid*
2.8	331.06703 (169)	-0.12	$C_{13}H_{16}O_{10}$	galloyl hexoside
3.4	169.01477	3.08	$C_7H_6O_5$	Gallic acid*
3.9	361.07735 (169,271)	-0.79	$C_{14}H_{18}O_{11}$	Galloyl-d-sedoheptulose
5.7	153.01974	2.65	$C_7H_6O_4$	Protocatechuic acid*
6.4	299.07782 (137)	1.93	$C_{13}H_{16}O_8$	hydroxybenzoic acid glucoside
7.3	311.04162 (179 , 149,135)	2.45	$C_{13}H_{12}O_9$	Caftaric acid* (cis/trans)
11.0	375.13017	1.33	$C_{16}H_{24}O_{10}$	Loganic acid*
11.20	311.04122 (179 , 149,135)	1.17	$C_{13}H_{12}O_9$	Caftaric acid* (cis/trans)
12.5	389.14589 (195,345)	1.46	$C_{17}H_{26}O_{10}$	Loganin
13.2	353.08843 (191,179)	1.76	$C_{16}H_{18}O_9$	Chlorogenic acid*
14.5	163.04011	0.26	$C_9H_8O_3$	p-coumaric acid*
16.3	447.09402 (284)	1.64	$C_{21}H_{20}O_{11}$	Cyanidin 3-O-galactoside*
16.9	337.09264 (191,173)	-0.74	$C_{16}H_{18}O_8$	p-coumaroylquinic acid
17.3	431.09891 (269)	1.25	$C_{21}H_{20}O_{10}$	Pelargonidin 3-O-glucoside
17.7	449.10994 (287)	2.23	$C_{21}H_{22}O_{11}$	Aromadendrin hexoside
18.2	337.09277	-0.36	$C_{16}H_{18}O_8$	p-coumaroylquinic acid
18.3	403.12492	0.83	$C_{17}H_{24}O_{11}$	Secoxyloganin
19.2	449.10994 (287)	2.23	$C_{21}H_{22}O_{11}$	Aromadendrin hexoside
20.8	449.10999 (269)	2.34	$C_{21}H_{22}O_{11}$	Unknown flavonoid
25.18	463.08864 (301)	0.95	$C_{21}H_{20}O_{12}$	Quercetin hexoside
25.2	477.06732 (301)	-0.3	$C_{21}H_{18}O_{13}$	Quercetin 3-glucuronide*
27.7	447.09195 (285)	-2.98	$C_{21}H_{20}O_{11}$	Kaempferol 3-O-galactoside
28.4	541.15887	4.78	$C_{24}H_{30}O_{14}$	Cornuside*

a

b

c

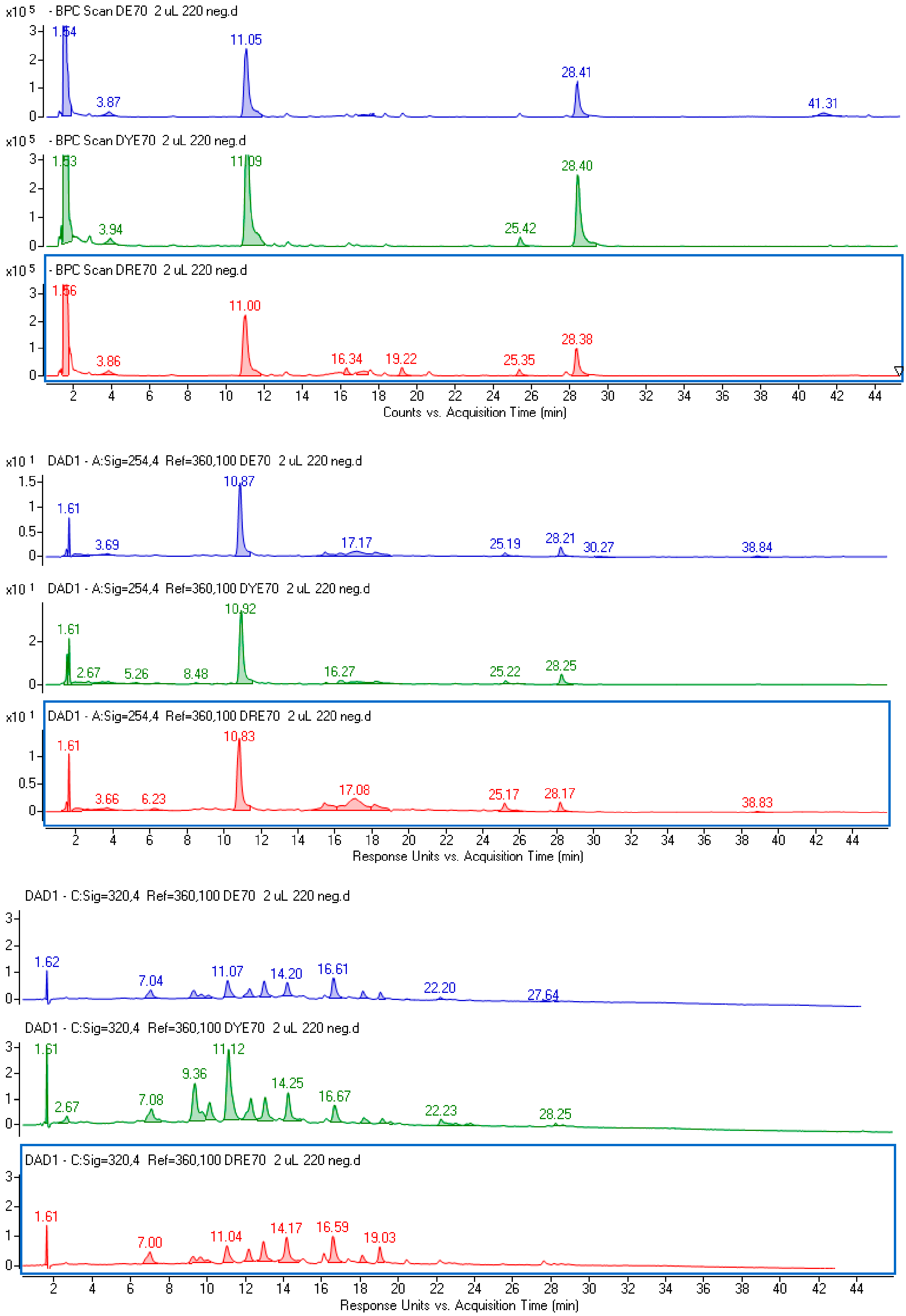

Figure S1. Representative chromatograms obtained for 70% ethanol extracts from red-fruit (blue line), yellow-fruit (green line) and dark ruby-red fruit (red line) of *C. mas* L. cultivars. **a** –

chromatograms recorded in negative ionization mode (BPC chromatogram); **b** – DAD chromatograms recorded at λ=254 nm; **c** - DAD chromatograms recorded at λ=320 nm

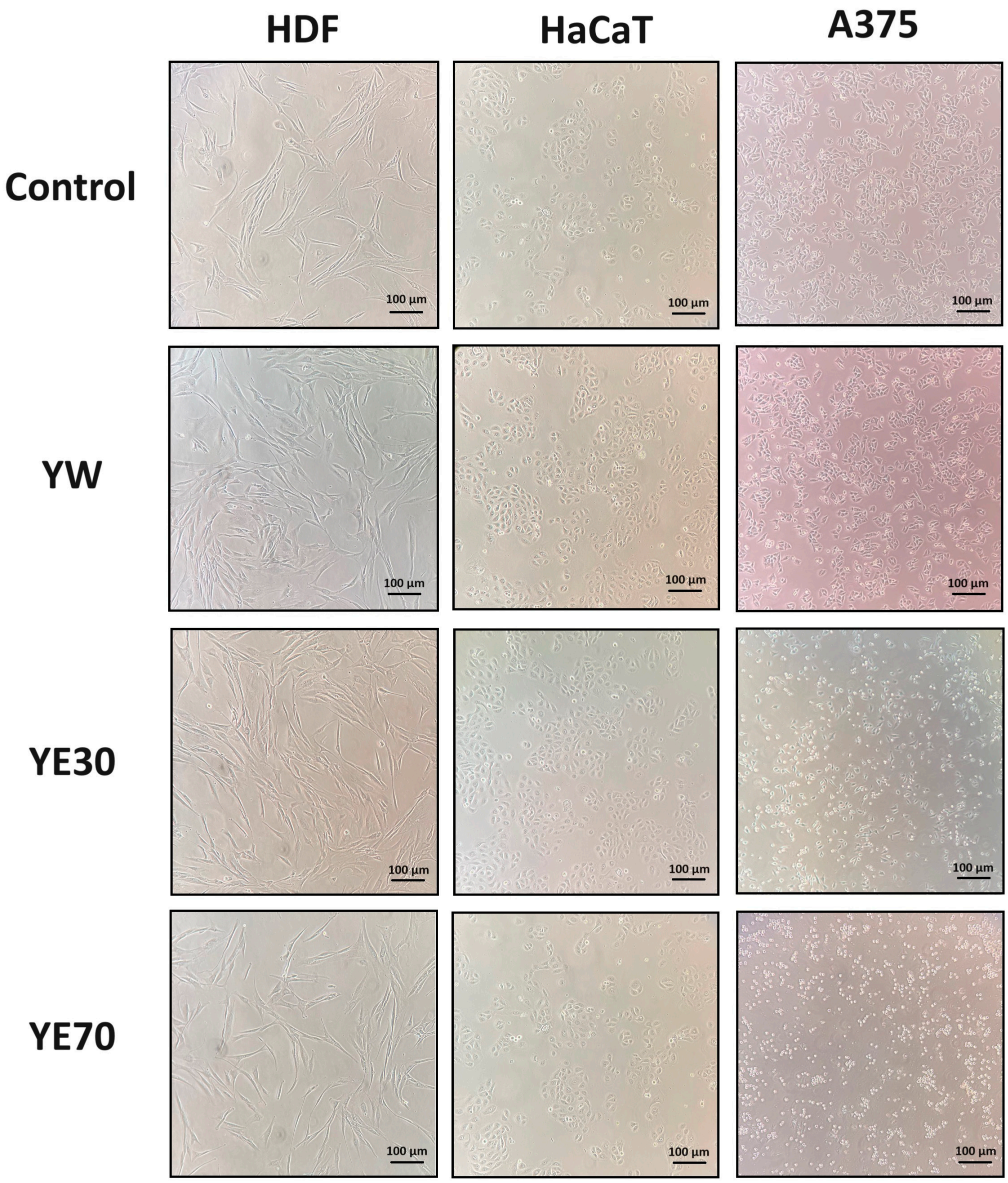

Figure S2. Microscopic images of fibroblasts (HDF), keratinocytes (HaCaT) and melanoma cells (A375) taken using an inverted fluorescence microscope. The photos show the morphology of cells exposed to both UVA radiation (5 J/cm^2 for 1 h) and three types of extracts (at a dilution of 1:10 (*v*/*v*)) from the yellow-fruited *C. mas* L. variety.

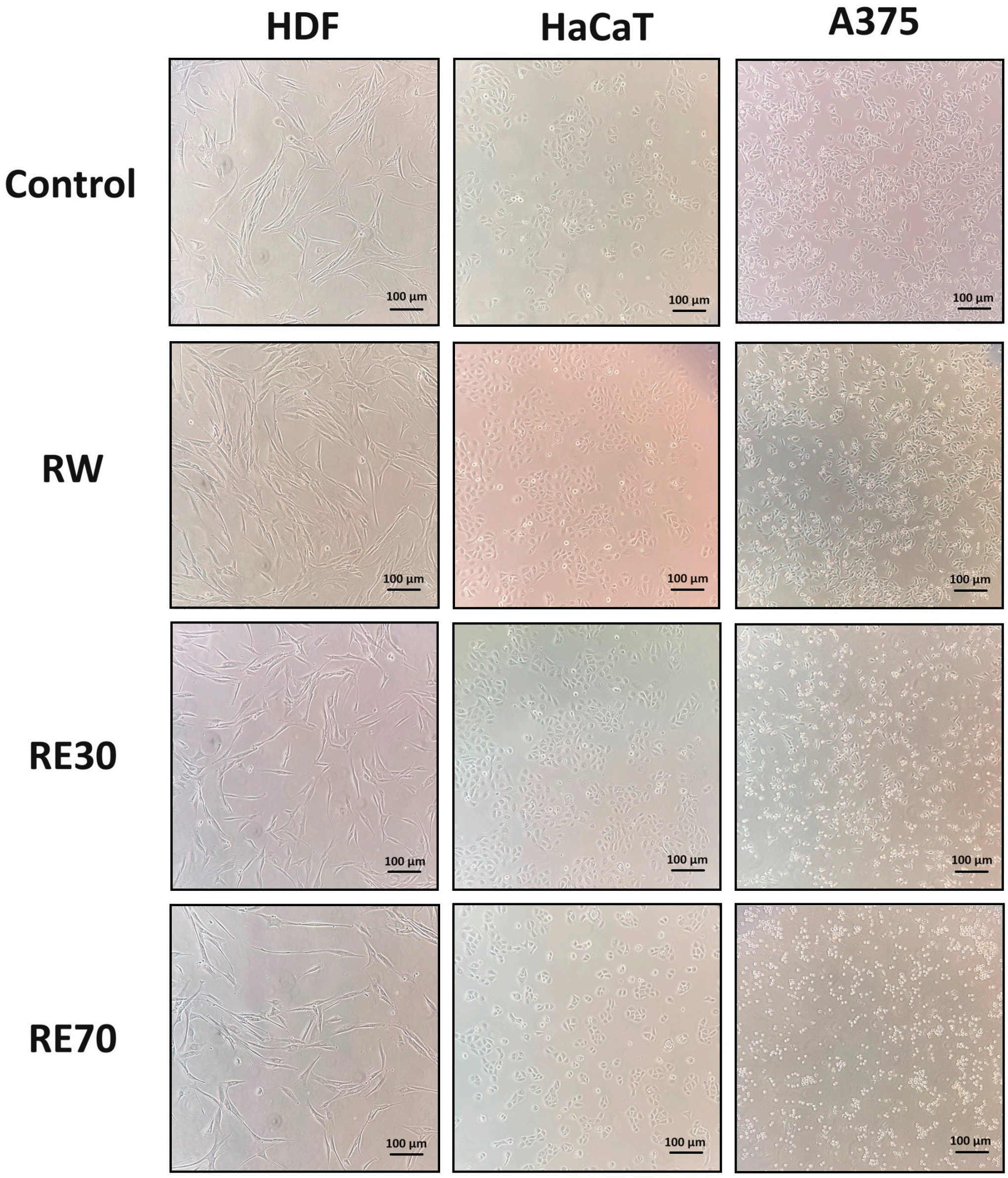

Figure S3. Microscopic images of fibroblasts (HDF), keratinocytes (HaCaT) and melanoma cells (A375) taken using an inverted fluorescence microscope. The photos show the morphology of cells exposed to both UVA radiation (5 J/cm^2 for 1 h) and three types of extracts (at a dilution of 1:10 (*v*/*v*)) from the red-fruited *C. mas* L. variety.

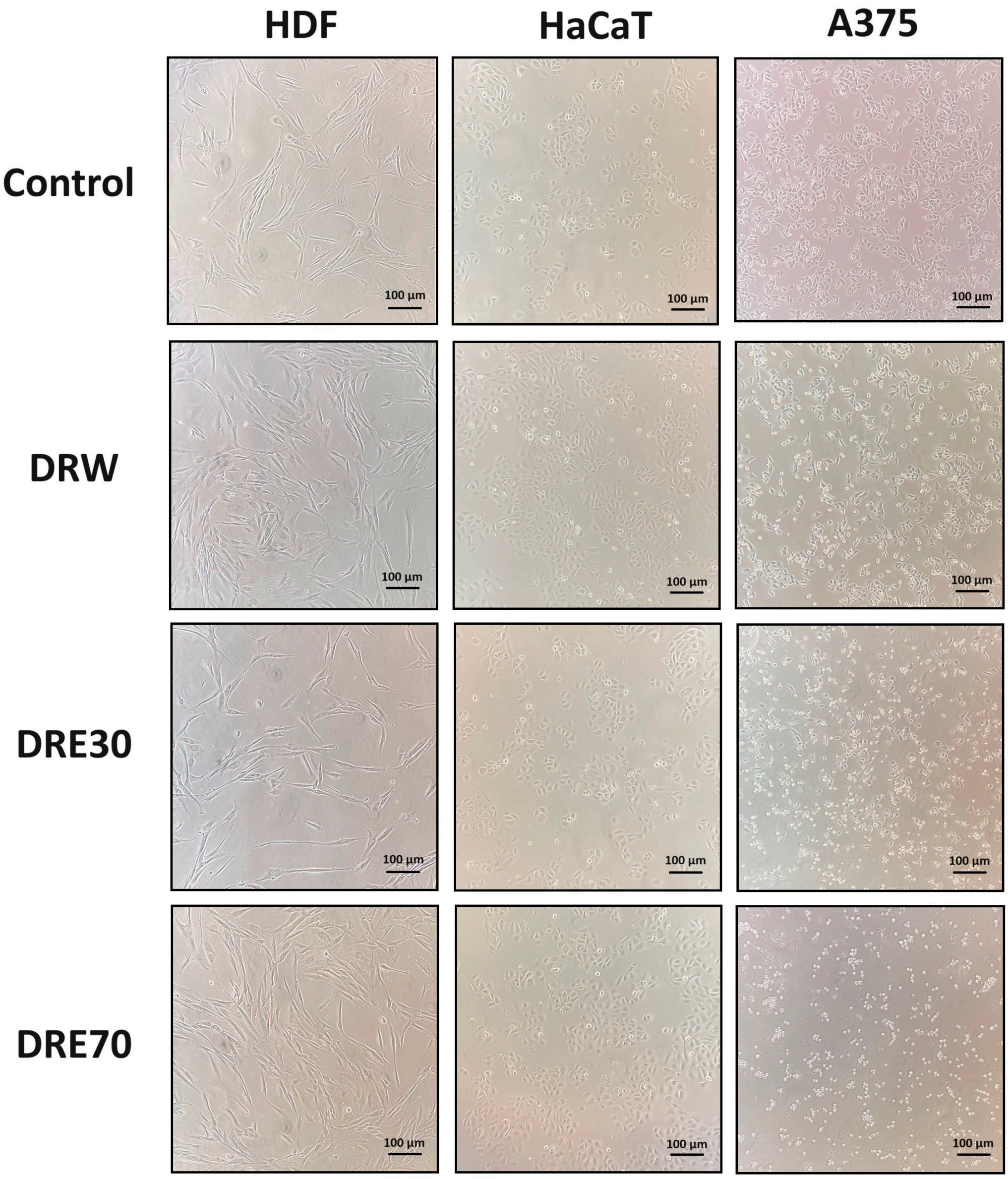

Figure S4. Microscopic images of fibroblasts (HDF), keratinocytes (HaCaT) and melanoma cells (A375) taken using an inverted fluorescence microscope. The photos show the morphology of cells exposed to both UVA radiation (5 J/cm2 for 1 h) and three types of extracts (at a dilution of 1:10 (*v*/*v*)) from the dark ruby red-fruited *C. mas* L. variety.

a
b
c

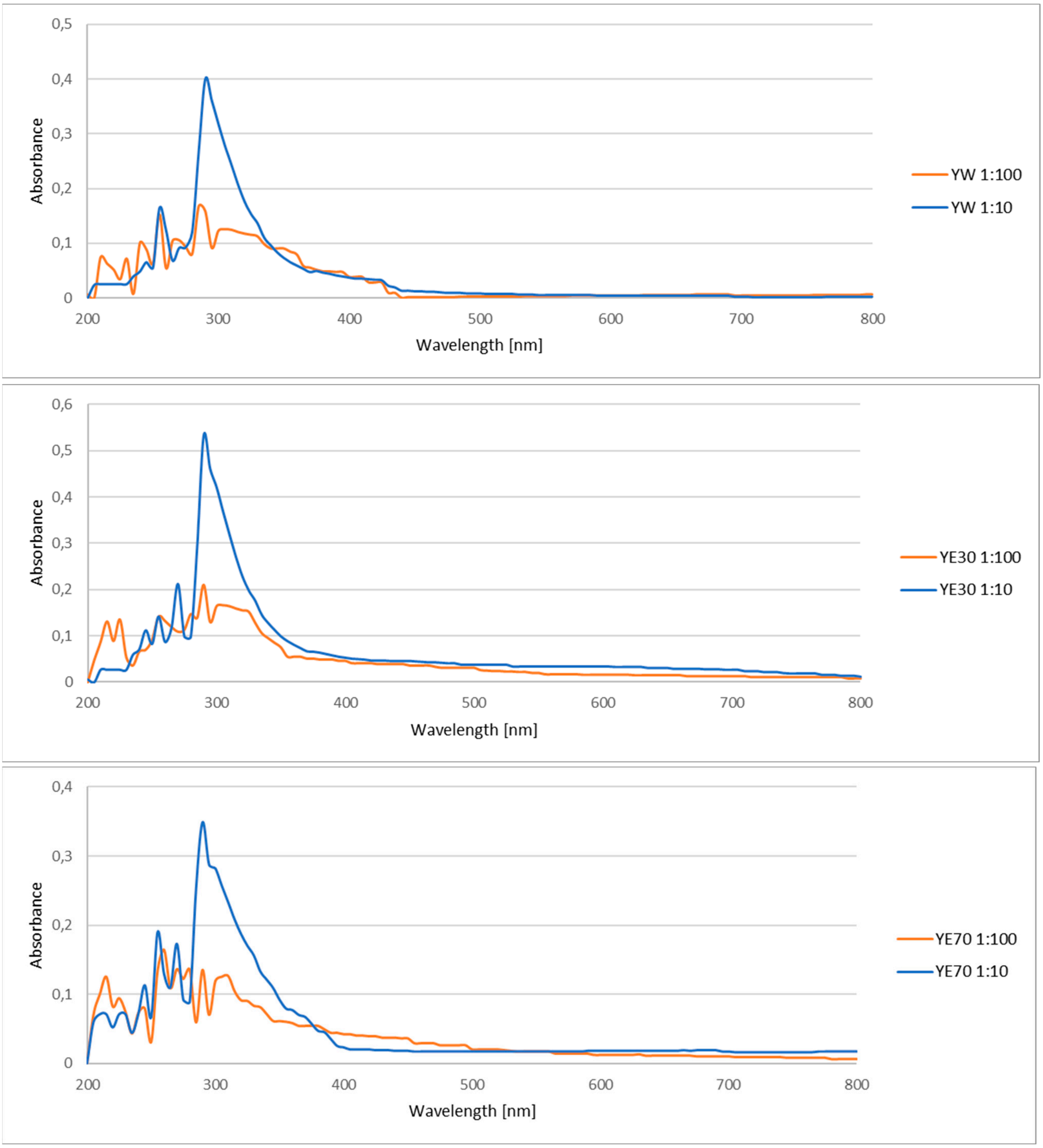
0,5
0,4
0,3
0,2
0,1
0
Absorbance
200 300 400 500 600 700 800
Wavelength [nm]
YW 1:100
YW 1:10
0,6
0,5
0,4
0,3
0,2
0,1
0
Absorbance
200 300 400 500 600 700 800
Wavelength [nm]
YE30 1:100
YE30 1:10
0,4
0,3
0,2
0,1
0
Absorbance
200 300 400 500 600 700 800
Wavelength [nm]
YE70 1:100
YE70 1:10

d

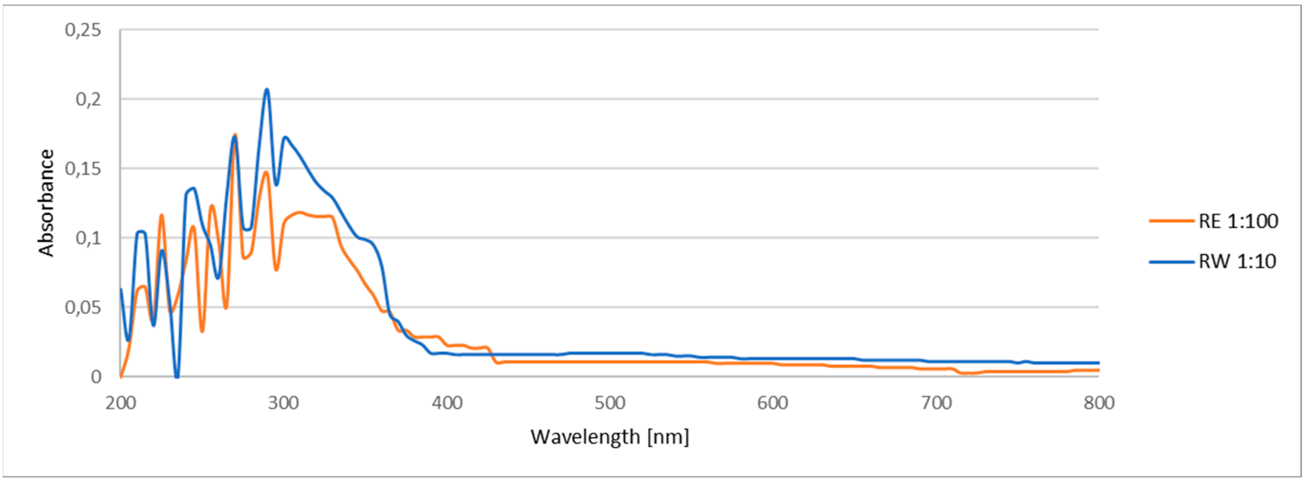
0,25
0,2
0,15
0,1
0,05
0
Absorbance
200
300
400
500
600
700
800
Wavelength [nm]
RE 1:100
RW 1:10

e

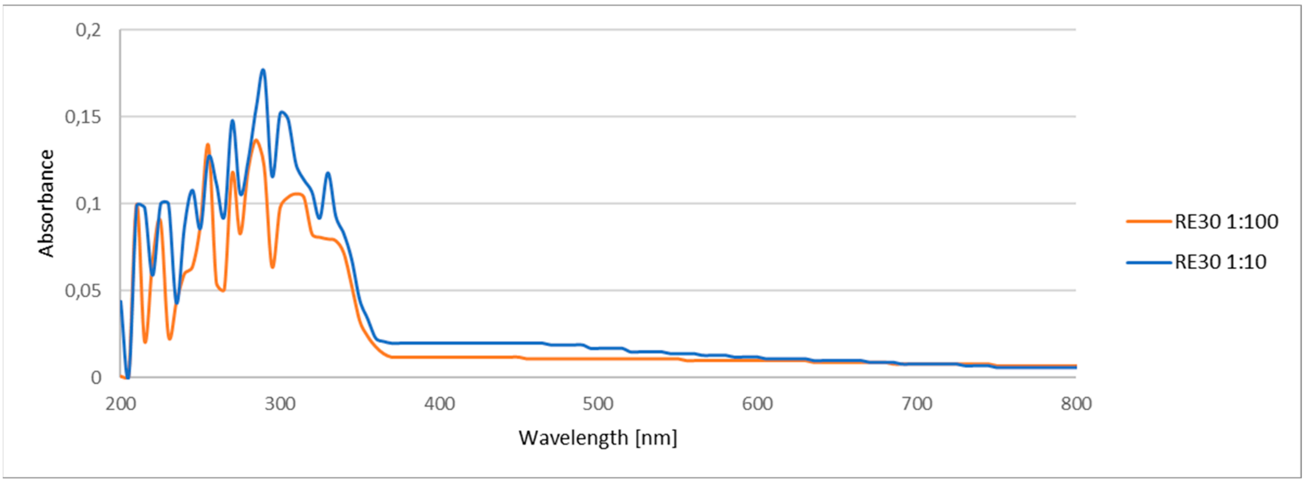
0,2
0,15
0,1
0,05
0
Absorbance
200
300
400
500
600
700
800
Wavelength [nm]
RE30 1:100
RE30 1:10

f

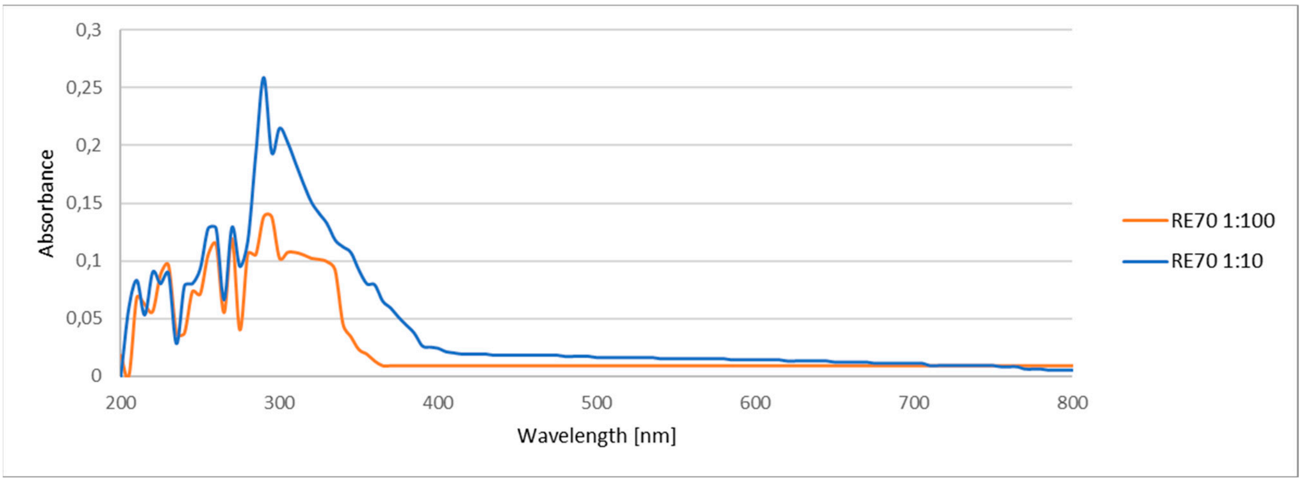
0,3
0,25
0,2
0,15
0,1
0,05
0
Absorbance
200
300
400
500
600
700
800
Wavelength [nm]
RE70 1:100
RE70 1:10

g

h

i

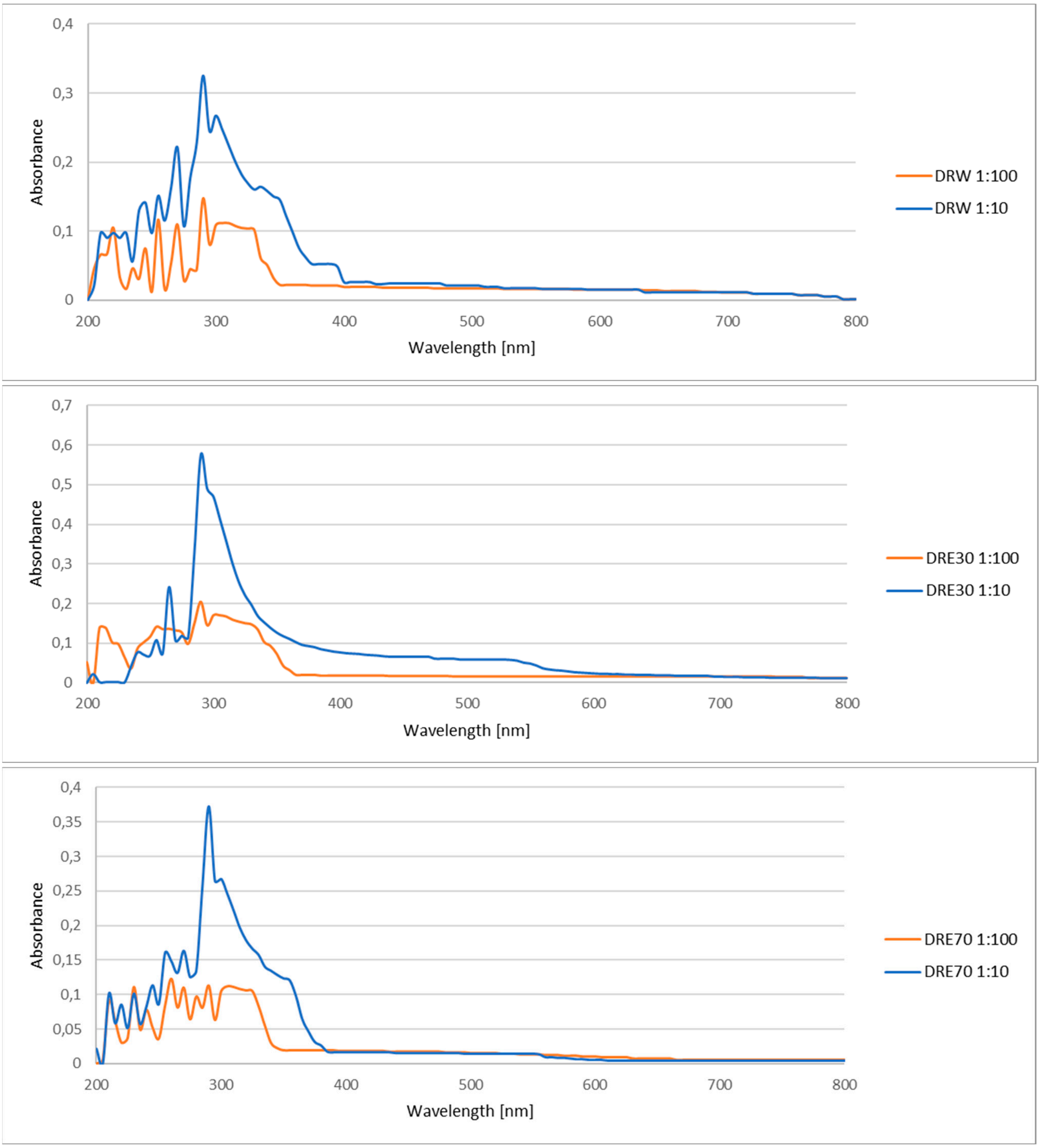

Figure S5. Absorption spectra for the tested extracts from *Cornus mas* L. Analyses were performed for extracts in dilutions of 1:100 and 1:10 (*v/v*). The figures represent: yellow fruit extracts (**a**: water (YW); **b**: water–ethanol 30:70 (YE30); **c**: water–ethanol 70:30 (YE70)), red fruit extracts (**d**: water (RW); **e**: water–ethanol 30:70 (RE30); **f**: water–ethanol 70:30 (RE70)) and dark ruby red extracts (**g**: water (DRW); **h**: water–ethanol 30:70 (DRE30); **i**: water–ethanol 70:30 (DRE70)).

a – YW ; b – YE30; c – YE70; d – RW; e – RE30; f – RE70; g – DRW; h – DRE30; i – DRE70
